# Vitamin B_5_ supports MYC oncogenic metabolism and tumor progression in breast cancer

**DOI:** 10.1038/s42255-023-00915-7

**Published:** 2023-11-09

**Authors:** Peter Kreuzaler, Paolo Inglese, Avinash Ghanate, Ersa Gjelaj, Vincen Wu, Yulia Panina, Andres Mendez-Lucas, Catherine MacLachlan, Neill Patani, Catherine B. Hubert, Helen Huang, Gina Greenidge, Oscar M. Rueda, Adam J. Taylor, Evdoxia Karali, Emine Kazanc, Amy Spicer, Alex Dexter, Wei Lin, Daria Thompson, Mariana Silva Dos Santos, Enrica Calvani, Nathalie Legrave, James K. Ellis, Wendy Greenwood, Mary Green, Emma Nye, Emma Still, CRUK Rosetta Grand Challenge Consortium, Paolo Inglese, Avinash Ghanate, Vincen Wu, Yulia Panina, Helen Huang, Gina Greenidge, Adam J. Taylor, Evdoxia Karali, Emine Kazanc, Amy Spicer, Alex Dexter, Peter Kreuzaler, Simon Barry, Richard J. A. Goodwin, George Poulogiannis, Greg McMahon, Zoltan Takats, Josephine Bunch, Mariia Yuneva, Simon Barry, Richard J. A. Goodwin, Alejandra Bruna, Carlos Caldas, James MacRae, Luiz Pedro Sório de Carvalho, George Poulogiannis, Greg McMahon, Zoltan Takats, Josephine Bunch, Mariia Yuneva

**Affiliations:** 1 The Francis Crick Institute, London, UK; 2 Faculty of Medicine, Department of Metabolism, Digestion and Reproduction, Imperial College London, South Kensington Campus, London, UK; 3 The National Physical Laboratory, Teddington, UK; 4 University of Cambridge, MRC Biostatistics Unit, Cambridge Biomedical Campus, Cambridge, UK; 5 Signalling and Cancer Metabolism Team, Division of Cancer Biology, The Institute of Cancer Research, London, UK; 6 University of Cambridge, Cancer Research UK Cambridge Institute, Li Ka Shing Centre, Cambridge, UK; 7 Imaging and Data Analytics, Clinical Pharmacology and Safety Sciences, R&D, AstraZeneca, Cambridge, UK; 8 Modelling of Paediatric Cancer Evolution, Centre for Paediatric Oncology, Experimental Medicine, Centre for Cancer Evolution: Molecular Pathology Division, The Institute of Cancer Research, Belmont, Sutton, London, UK; 9 The Rosalind Franklin Institute, Harwell Campus, Didcot, UK; 10 Present Address: University of Cologne, Faculty of Medicine and University Hospital Cologne, Cluster of Excellence Cellular Stress Responses in Aging-associated Diseases (CECAD), Cologne, Germany; 11 Present Address: Department of Physiological Sciences, University of Barcelona, Barcelona, Spain

**Keywords:** Cancer, Breast cancer, Metabolism

## Abstract

Tumors are intrinsically heterogeneous and it is well established that this directs their evolution, hinders their classification and frustrates therapy^1–3^. Consequently, spatially resolved omics-level analyses are gaining traction^4–9^. Despite considerable therapeutic interest, tumor metabolism has been lagging behind this development and there is a paucity of data regarding its spatial organization. To address this shortcoming, we set out to study the local metabolic effects of the oncogene *c-MYC*, a pleiotropic transcription factor that accumulates with tumor progression and influences metabolism^10,11^. Through correlative mass spectrometry imaging, we show that pantothenic acid (vitamin B_5_) associates with MYC-high areas within both human and murine mammary tumors, where its conversion to coenzyme A fuels Krebs cycle activity. Mechanistically, we show that this is accomplished by MYC-mediated upregulation of its multivitamin transporter SLC5A6. Notably, we show that SLC5A6 over-expression alone can induce increased cell growth and a shift toward biosynthesis, whereas conversely, dietary restriction of pantothenic acid leads to a reversal of many MYC-mediated metabolic changes and results in hampered tumor growth. Our work thus establishes the availability of vitamins and cofactors as a potential bottleneck in tumor progression, which can be exploited therapeutically. Overall, we show that a spatial understanding of local metabolism facilitates the identification of clinically relevant, tractable metabolic targets.

## Abstract (web summary)

In this study, Kreuzaler et al. perform zonal analysis to study metabolic heterogeneity in breast cancer and identify the metabolic dependency on pantothenic acid (vitamin B5) in areas of the tumor that show high expression levels of the oncogene MYC. Dietary restriction of vitamin B5 reverses several MYC-driven metabolic changes and hampers tumor progression.

## Main

All tumors, including breast cancers, show profound clonal heterogeneity^12^. These clones in turn have to adapt to the ever-changing tumor microenvironment, frequently characterized by fluctuating oxygen and nutrient supply^13^. This requires tumor cells to constantly rewire their metabolic pathways and consequently many tumors show a high degree of metabolic flexibility^14,15^. Intrinsic oncogene-driven metabolic programs are thus integrated with environmental cues to shape the net local metabolism of tumor cells.

MYC is a proto-oncogene and a pleiotropic transcription factor, and clones with high MYC expression levels arise as subclones during malignant progression in a range of cancers, and generally correlate with higher grade and poor survival^3,10^. While MYC is recognized as a master regulator of metabolism, inducing glycolytic flux and increasing glutaminolysis among others^11,16,17^, the true metabolic signature of these malignant subclones in the pathophysiologically relevant context of multiclonality remains unknown. Conversely, unveiling the metabolic traits of MYC-high clones in situ would enable us to devise new therapeutic strategies targeted at the metabolism underlying malignant tumor progression.

## In situ segmentation of multiclonal mammary tumors

**Extended Data Fig. 1:**
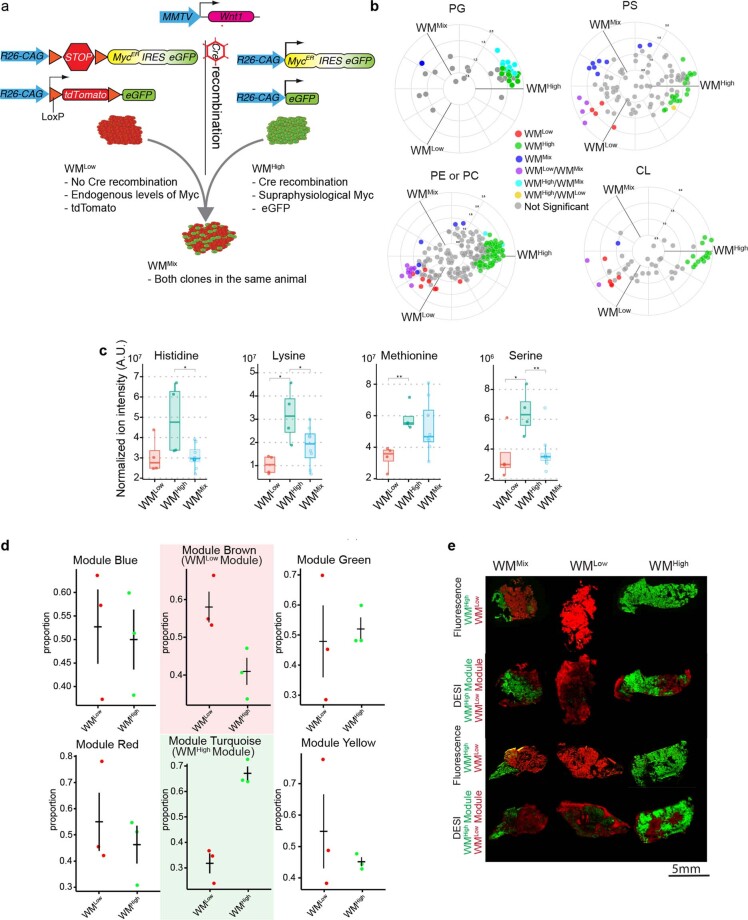
WM tumors were analyzed with LC–MS analysis and spatial metabolomics by DEFFI. **a**, Overview of the transgenic background of the three WM tumors subtypes. **b**, Lipid classes from the apolar fraction of WM^High^, WM^Low^ and WM^Mix^ tumors analyzed with LC–MS were selected based on their association with WM^High^ and plotted on a radial plot using the R package volcano3D. The radial angle ρ represents relative affiliation of metabolites to the individual samples and the distance from center - the relative amounts. Colors indicate statistically significant affiliation to one or two samples (PG: phosphatidylglycerol, PS: phosphatidylserine, PE: phosphatidylethanolamine, PC: phosphatidylcholine, CL: cardiolipin). Significance was calculated with an unpaired two-tailed *t*-test (WM^High^, n = 4; WM^Low^, n = 4; WM^Mix^, n = 8 tumors from independent animals). **c**, Selected metabolites from the LC–MS analysis of the polar fractions of WM^High^, WM^Low^ and WM^Mix^ were plotted, showing increased levels of amino acids in WM^High^ tumors. 10 mg of dry tissue from each sample were taken for the extraction. Significance was calculated with an unpaired two-tailed *t*-test (WM^High^, n = 4; WM^Low^, n = 4; WM^Mix^, n = 8. Histidine: WM^High^ vs WM^Mix^, *P* = 0.02084; Lysine: WM^High^ vs WM^Low^, *P* = 0.0135; WM^High^ vs WM^Mix^, *P* = 0.0448; Methionine: WM^High^ vs WM^Low^, *P* = 0.0044; Serine: WM^High^ vs WM^Low^, *P* = 0.0445; WM^High^ vs WM^Mix^, *P* = 0.0095). **d**, Dot-plots represent the proportion of pixels in the WM^Low^ and WM^High^ tumors respectively with intensities higher than the median in the module eigenmetabolite. The graphs show that the Brown module is more prevalent in the WM^Low^ tumors, while the Turquoise module correlates with the WM^High^ tumors. Data represents tree independent biological replicates (error bars: confidence interval). **e**, Post DEFFI fluorescent microscopy of WM^High^, WM^Low^ and WM^Mix^ tumors is shown and ion colocalization analysis of DEFFI acquired images of WM^High^, WM^Low^ and WM^Mix^ tumors reveals a WM^High^ module (green) and a WM^Low^ module (red). These are the two additional runs to the ones shown in Fig. 1f. *P* value: *<0.05, ** 0.001, ***<0.0001, ****<0.00001. See also Fig. 1.

To address this problem, we initially resorted to an inducible and traceable model of MYC heterogeneity in breast cancer, which we had previously developed and characterized^18^. In brief, this model allows us to create triple-negative mammary tumors, driven by WNT1, but optionally also containing a MYC-ER^T2^ construct, which expresses supraphysiological levels of the MYC-ER fusion protein and is activated by administration of tamoxifen. We term the tumors WM, reflecting the two oncogenes involved. The clones without MYC-ER^T2^ (WM^low^) express tdTomato as a tracer, whereas the ones with MYC-ER^T2^ (WM^high^) express enhanced green fluorescent protein (eGFP). Mixing the two clones generates biclonal tumors (WM^mix^) (Extended Data Fig. 1a). Throughout the study, MYC-ER^T2^ activation was performed acutely for a duration of only 3 d unless otherwise stated.

**Fig. 1:**
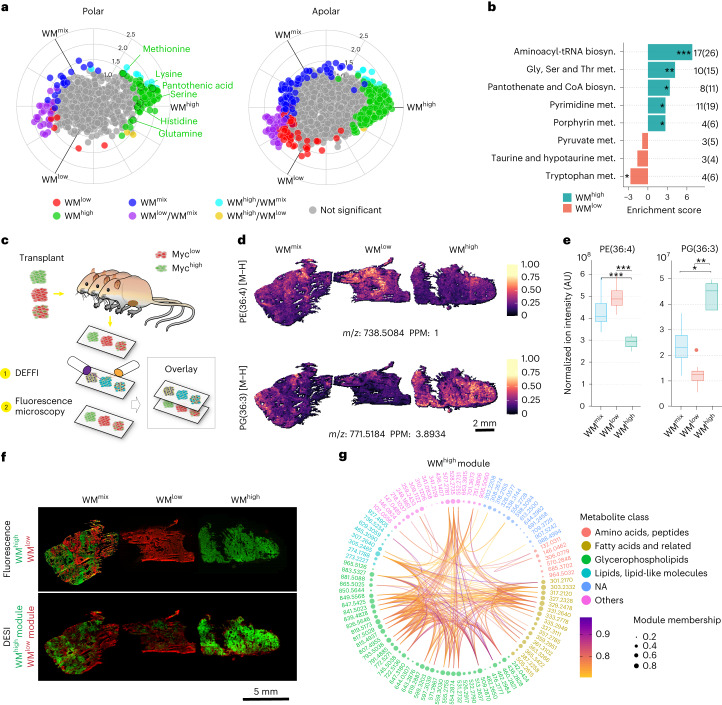
In situ segmentation of multiclonal mammary tumors. **a**, Polar and apolar fractions of WM^high^, WM^low^ and WM^mix^ tumors were analyzed with LC–MS (10 mg dry tissue from each sample was taken for the extraction) and plotted on a radial plot using the R package volcano3D. The radial angle ρ represents relative affiliation of metabolites to the individual samples and the distance from center the relative amounts. Colors indicate statistically significant affiliation to one or two samples. Significance was calculated with an unpaired two-tailed *t*-test (WM^high^, *n* = 4; WM^low^, *n* = 4; WM^mix^, *n* = 8 tumors from independent animals). **b**, Metabolic pathways analysis of the data in **a** showing several significantly changed pathways and numbers of identified members against the pathway size. **c**, Schematic of tissue processing and DEFFI imaging. **d**,**e**, Levels and distribution of two selected ions as measured by DEFFI (**d**) or LC–MS (**e**) show concordance between the methodologies. Significance was calculated with an unpaired two-tailed *t*-test (WM^high^, *n* = 4; WM^low^, *n* = 4; WM^mix^, *n* = 8 tumors from independent animals (WM^high^ versus WM^low^, *P* = 0.00234; WM^high^ versus WM^mix^, *P* = 0.0089 (left); WM^high^ versus WM^low^, *P* = 0.0047; WM^high^ versus WM^mix^, *P* = 0.0154 (right)). **f**, Post-DEFFI fluorescent microscopy of WM^high^, WM^low^ and WM^mix^ tumors show clonal distribution (top). Ion colocalization analysis of DEFFI acquired images of WM^high^, WM^low^ and WM^mix^ tumors reveal a WM^high^ module (green) and a WM^low^ module (red) (bottom). **g**, Circos plot representing all metabolites found in the WM^high^ module. Nodes represent the metabolites in the module. Node size is proportional to module membership, which represents a given metabolite’s strength of the colocalization index and is calculated as Pearson’s correlation between the spatial intensities of the corresponding metabolite and the module’s eigenmetabolites. Arc lines connect node pairs corresponding to colocalized metabolites with Pearson’s correlation >0.7. The nodes are color-grouped according to metabolite class based on Human Metabolome Database putative annotations (|*m*/*z* error| < 10 ppm). All box-and-whisker plots represent the following: line, median; box, interquartile range (IQR); whiskers, 1.5 × IQR limited by largest/smallest non-extreme value (NEV). In all DEFFI-MSI experiments *n* = 3 tumors from independent animals for each WM tumor type. *P* values indicated by *<0.05, ** 0.001, ***<0.0001, ****<0.00001. Source data

Metabolomic characterization of the three WM tumor types confirmed previous observations seen when acutely switching on MYC in tumors, including an increase in the levels of several amino acids, as well as phosphatidylethanolamine (PE)/phosphatidylcholine (PC) and phosphatidylglycerol (PG) (Fig. 1a and Extended Data Fig. 1b,c)^17,19^. Metabolic pathway analysis of WM^high^ tumors showed significant increases in pathways related to cellular growth, including serine and glycine metabolism, pyrimidine biosynthesis as well as aminoacyl-tRNA biosynthesis (Fig. 1b). With few exceptions, the metabolite levels found in WM^mix^ tumors range in between the ones from the pure clonal tumors (Fig. 1a and Extended Data Fig. 1c), reflecting the partial contribution of the individual clonal populations. This highlights the difficulty in analyzing bulk tissue data of multiclonal tumors and the need for spatially resolved metabolomic data to disentangle the individual metabolic contributions and find possible vulnerabilities of the constituent clones.

We thus set out to define the metabolites and metabolic pathways most closely associated with the individual WM clones in situ. To this end we devised a strategy of multimodal correlative imaging that combined desorption electro-flow focusing ionization (DEFFI)^20^ mass spectrometric imaging (MSI) with fluorescence microscopy (Fig. 1c). Comparing metabolites co-detected in liquid chromatography–mass spectrometry (LC–MS) and DEFFI-MSI, we saw a good concordance between the methods for a number of metabolites with regards to their respective distribution in the WM^high^ and WM^low^ tumors (for example Figure 1d,e). Notably, the WM^mix^ tumors frequently showed a pattern of zonation, which followed their clonal landscape (Fig. 1d,f). To segment the tumors in an unbiased way, we adopted a recently published method of ion colocalization analysis that combines groups of ions into so-called modules based on their similar spatial behavior throughout the entire acquired image^21,22^. We identified six ion modules, of which, based on their distribution, one correlated with WM^high^ (module ‘Turquoise’) and one with WM^low^ (module ‘Brown’) tumors (Fig. 1f, Extended Data Fig. 1d,e and Supplementary Table 1). The two modules, once projected onto the tumor tissues of the WM^mix^ tumors, reflect the clonal landscape, highlighting the clonal specificity of our automated segmentation. Comparing the molecular features in the identified modules, we noticed that in the WM^high^ module, in accordance with our data from the bulk analysis (Extended Data Fig. 1b), PGs and PE/PCs constituted the largest group of metabolites and that there was significant spatial correlation within, as well as between compound classes, a number of which were validated via tandem MS (MS/MS) in both methodologies (Fig. 1g (connecting lines), Supplementary Table 1 and Extended Data Table 1). Furthermore, in keeping with the literature, eicosanoids represented a prominent lipid class in the WM^high^ module^23^. Overall, these data underscore the robustness of our imaging and segmentation approach.

## Pantothenic acid correlates with MYC-high tumor areas

**Fig. 2:**
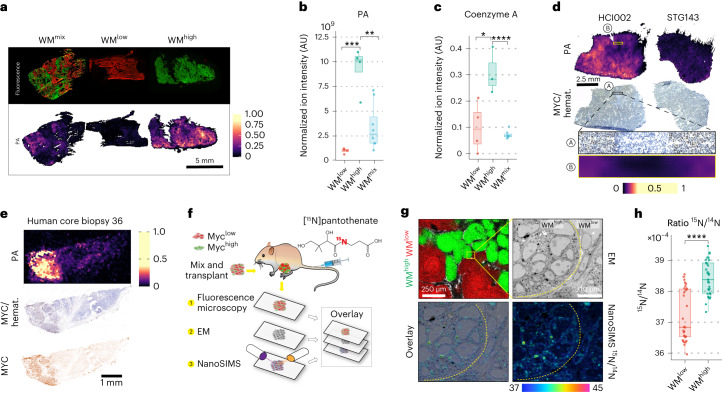
Pantothenic acid correlates with MYC expression in mammary tumors. **a**, Post-DEFFI fluorescent microscopy of WM^high^, WM^low^ and WM^mix^ tumors shows clonal distribution (note, this is the same image as Fig. 1f, repeated for illustrative purposes) (top). Single-ion DEFFI image of PA (*n* = 3 for each tumor type) (bottom). **b**, LC–MS analysis of WM tumors (10 mg dry tissue from each sample was taken for the extraction) shows significant increase of PA in WM^high^ tumors (WM^high^ versus WM^low^, *P* = 0.00038; WM^high^ versus WM^mix^, *P* = 0.0019). **c**, LC–MS analysis of free CoA-SH in WM tumors shows an increase in WM^high^ tumors (WM^high^, *n* = 4; WM^low^, *n* = 4; WM^mix^, *n* = 8 tumors from independent animals; WM^high^ versus WM^low^, *P* = 0.0313; WM^high^ versus WM^mix^, *P* = 7.68 × 10^−5^) (**b**,**c**). **d**, Correlative DEFFI and IHC staining showing the distribution of PA in relation to MYC staining in human PDXs (*n* = 2 biological replicates for each PDX). **e**, Correlative DEFFI and IHC staining showing the distribution of PA in relation to MYC staining in representative human core biopsy, showing association of MYC with PA (*n* = 12 core biopsies). **f**, Workflow for correlative fluorescence microscopy, EM and NanoSIMS analysis. **g**, Correlative fluorescence microscopy, EM and NanoSIMS analysis shows ^15^N derived from ^15^N-PA infusions predominantly localizing in MYC-high cells, with subcellular localization in mitochondria and nucleoli. **h**, Cell-wise quantification of ^15^N/^14^N ratios in the WM^high^, WM^low^ areas shows increased amounts of PA-derived labeled ^15^N in WM^high^ cells. For NanoSIMS 34 WM^high^ and 32 WM^low^ individual cells were analyzed from one NanoSIMS run (*P* = 2.91 × 10^−9^). All box-and-whisker plots represent the following: line, median; box, IQR; whiskers, 1.5 × IQR limited by largest/smallest NEV. Significance was calculated with an unpaired two-tailed *t*-test. *P* values are represented by *<0.05, ** 0.001, ***<0.0001, ****<0.00001. Source data

**Extended Data Fig. 2:**
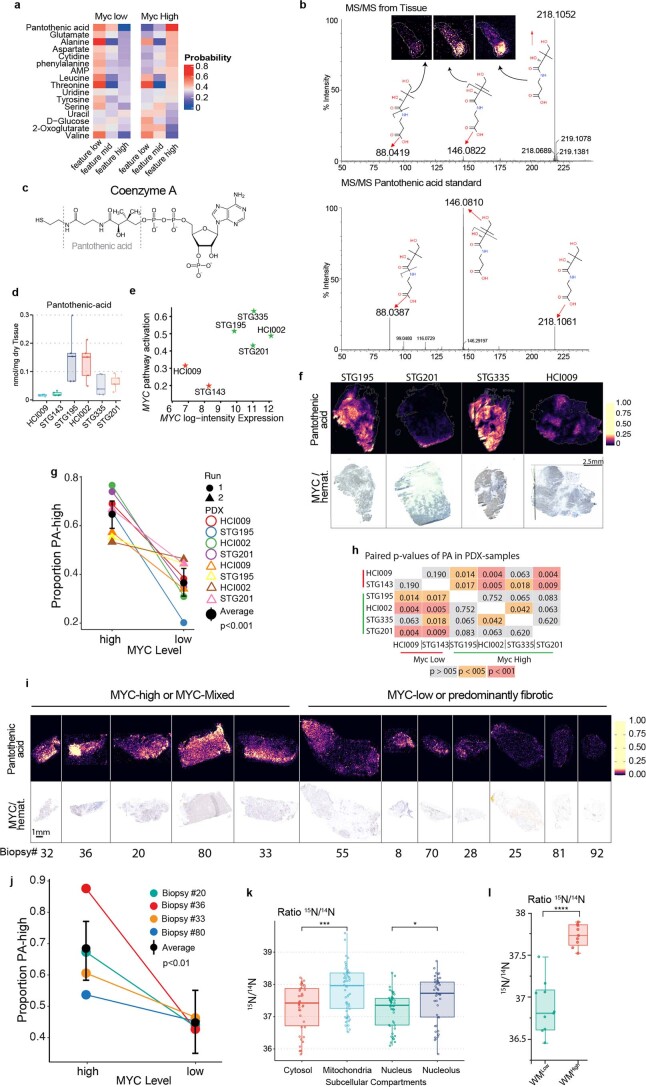
PA correlates with MYC expression in mammary tumors. **a**, Conditional probabilities of pixels with low, medium and high amounts of the metabolites specified were calculated for WM^low^ and WM^High^ tumors, showing a very high association of high levels of PA with WM^High^ tumors. **b**, MS/MS spectra for confirmation of PA using *in situ* tandem MS fragmentation of the PA precursor ion both from a standard applied to the slide prior to imaging and from primary tumor tissue (HCI002). Identical mass peaks as well as very similar relative peak intensities confirm the primary peak as PA. **c**, Chemical structure of CoA highlighting the part constituted by pantothenic acid. **d**, GC–MS analysis for PA content in human PDXs (n = 5 tumors from independent animals for each PDX). **e**, *MYC* expression and *MYC* pathway activation of analyzed PDXs were plotted and show good general concordance between *MYC* expression and pathway activation. **f**, Correlative DEFFI and IHC staining showing the distribution of PA in relation to MYC staining in human PDXs (n = 2 biological replicates for each PDX). **g**, Proportion of pixels with higher than median levels of PA in MYC high and MYC low areas measured by DEFFI on PDX samples. Data was extracted by automated segmentation on immunostaining for MYC and the masks were projected onto the measurements acquired in DEFFI. Areas of overt necrosis and any staining and processing artefacts were excluded from the quantification (Supplementary Figs. 1 and 2). The average difference of the proportion of high levels of PA in areas high or low for MYC respectively was found significantly different from zero by a linear mixed effect model fitted with the ‘glmmTMB‘ package for R (random intercepts were considered for run and tissue ID). The algorithm was applied for all data from two independent biological repeats of 4 independent PDXs. Solid black dot represents the average of all runs. Error bar represents the confidence interval. **h**, Pairwise *P* values of an unpaired two-sided Student’s *t*-test performed on the GC–MS analysis of PA in PDX samples as plotted in Extended Data Fig. 2e shows higher similarity between samples with the same *MYC* activation status. **i**, Correlative DEFFI and IHC staining showing the distribution of PA in relation to MYC staining in human core biopsies, showing association of MYC with PA (n = 12 core biopsies). **j**, Proportion of pixels with higher than median levels of PA in MYC high and Myc low areas measured by DEFFI on human core biopsies. Data was extracted by automated segmentation on immunostaining for MYC and these masks were projected onto the measurements acquired in DEFFI. Areas of overt necrosis and any staining and processing artefacts were excluded from the quantification (Supplementary Fig. 3). The analysis was performed as described in **(g)**. The algorithm was applied on 4 independent human core biopsies. Solid black dot represents the average of all samples. Error bar represents the confidence interval. **k**, Guided by the EM, tumors were divided into subcellular compartments. The ROI was applied to NanoSIMS measurements and shows ^15^N derived from ^15^N-PA infusions predominantly localizes to the mitochondria (cytosol vs. mitochondria, p = 0.0005). Within the nuclear compartment, the nucleolus shows more label incorporation (nucleus vs nucleolus, p = 0.0124). Data represents one technical repeat of one sample. **l**, Quantification of ^15^N/^14^N ratios in randomly selected WM^High^, WM^Low^ areas shows increased amounts of ^15^N-PA-derived ^15^N in WM^High^ areas. This is a biological replicate confirming the results in Fig. 2g, h. Significance within this technical repeat was calculated with an unpaired two-tailed *t*-test (*P* = 6.066e^−7^). All box and whisker plots represent the following: Line: median, box: IQR, whiskers: 1.5x IQR limited by largest/smallest NEV. *P* value: *<0.05, ** 0.001, ***<0.0001, ****<0.00001. See also Fig. 2 and Appendix Figs. 1, 2, and 3. Source data

To identify metabolites that are central to Myc-mediated metabolic rewiring, we reasoned that a combination between the bulk metabolic analysis and the MSI data would give us the most relevant candidates. We thus extracted all statistically significantly changed metabolites identified by the bulk metabolic pathways analysis (Fig. 1b) and interrogated how well they correlated with the WM^high^ and WM^low^ tumors as well as their affiliation to the WM^high^ associated module ‘Turquoise’. By far the strongest correlation was observed for pantothenic acid (vitamin B5; henceforth PA), the chemical structure of which was confirmed by MS/MS using both a chemical standard and tumor tissues (Extended Data Fig. 2a,b). PA is the precursor for coenzyme A (CoA), a thiol that serves as an activator of carboxylic groups by forming thioesters (Extended Data Fig. 2c). As such, it is required for a number of key metabolic pathways, including the Krebs cycle and both fatty acid biosynthesis and oxidation^24,25^. Of note, in our correlative MSI, PA had a high overlap with the WM^high^ clones in the WM^mix^ tumors (Fig. 2a), which also coincided with an upregulation of PA as well as its main downstream product, CoA-SH, in WM^high^ tumors as measured by bulk LC–MS analysis (Fig. 2b,c). We thus decided to further study the involvement of the PA–CoA axis in MYC-mediated metabolism.

To investigate the clinical relevance of this finding, we first used a panel of human patient-derived xenografts (PDXs) subcutaneously transplanted into immunocompromised mice^26^. PDXs with the highest MYC levels or a strong *MYC* transcriptional profile showed a robust increase in PA levels (Extended Data Fig. 2d,e). To further understand the relationship between MYC expression and PA in situ, we adapted the aforementioned correlative MSI approach to combine DEFFI imaging with immunohistochemistry (IHC). Indeed, correlative MSI confirmed that intertumorally and intratumorally, areas of high MYC displayed increased levels of PA (Fig. 2d, Extended Data Fig. 2f–h and Supplementary Fig. 2). Notably, DEFFI revealed that higher signal intensity of PA also strongly correlated with areas of high MYC expression in primary human breast cancer biopsies (Fig. 2e, Extended Data Fig. 2i,j and Supplementary Fig. 3) supporting the clinical significance and the general validity of our observed connection between MYC and PA metabolism.

Increased levels of metabolites as seen in WM^high^ areas can be a cell-autonomous event, or in some cases they can be governed by field effects, such as a better overall vascularization due to MYC-mediated angiogenesis^18^. To ascertain that the accumulation of PA in WM^high^ areas of tumors is indeed driven by the WM^high^ cells, we devised a new, ultra-high-resolution, correlative MSI technique that combines fluorescence microscopy, electron microscopy (EM) and nanoscale secondary ion mass spectrometry (NanoSIMS)^27^, allowing for subcellular localization of metabolite incorporation (Fig. 2f). After administration of [^13^C_3_,^15^N]PA, the fluorescence image of the WM^mix^ sections was overlaid with the respective EM and NanoSIMS images (Fig. 2g). As expected, mitochondria, which harbor about 70% of a cell’s CoA (cellular concentration 10–50 µM)^28^, were clear hotspots of PA-derived ^15^N incorporation, but also the nucleoli showed a substantial signal, which is consistent with recent reports of significant HDAC activity in nucleoli (Extended Data Fig. 2k)^29^. Notably, WM^high^ cells incorporated significantly more PA-derived ^15^N compared to WM^low^ cells, strongly suggesting that the increased levels of PA observed in WM^high^ areas of WM^mix^ tumors are due to a cell-autonomous increase in uptake of PA, which is then used to synthesize downstream metabolites such as CoA (Fig. 2h and Extended Data Fig. 2l).

## MYC-high areas expand the pools of Krebs cycle intermediates

**Fig. 3:**
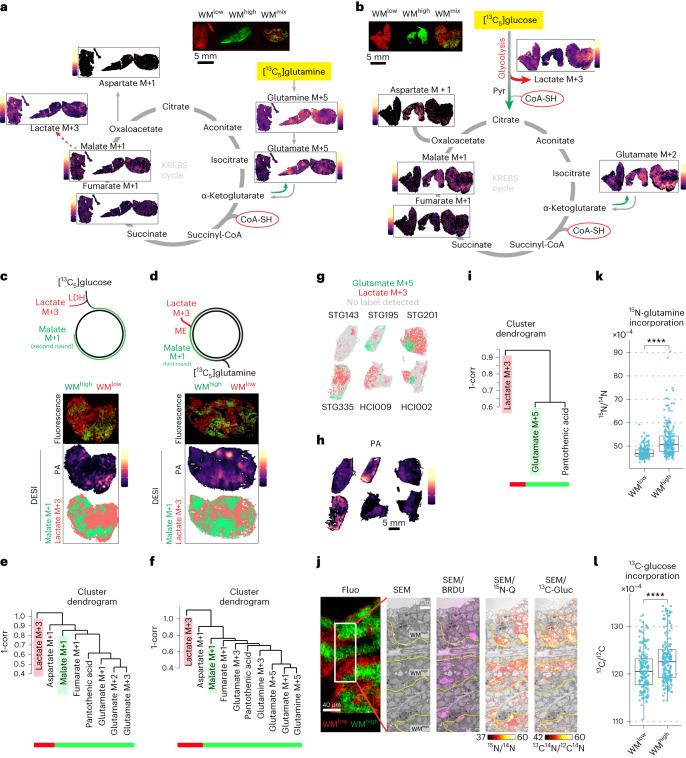
Pantothenic acid correlates with areas of high MYC and anticorrelates with lactagenic metabolism. **a**,**b**, DEFFI analysis of label incorporation into selected metabolites of WM tumors post infusion with [^13^C_5_]glutamine (**a**) or [^13^C_6_]glucose stripped for the natural abundance (**b**). **c**,**d**, Schematic of possible routes of ^13^C label incorporation after [^13^C_6_]glucose (**c**), or [^13^C_5_]glutamine (**d**) infusion (top). Post-DEFFI fluorescent microscopy of WM^mix^ tumors (bottom). Binarized representation of the labeled proxy compounds (lactate M+3 isotopologue for lactagenic metabolism and malate M+1 isotopologue for increased Krebs cycle) showing Krebs cycle activity corresponds with higher PA and WM^high^ areas (bottom). **e**,**f**, Dendrogram clustering between indicated proxy compounds and PA in [^13^C_6_]glucose (**e**) or [^13^C_5_]glutamine (**f**) infused tumors show a correlation for Krebs cycle proxies and no correlation with lactate. **g**–**i**, Binarized images for glutamate M+5 isotopologue and lactate M+3 isotopologue (**g**) in six human PDXs shows less labeled lactate in areas of increased PA (**h**), with a positive correlation of PA with the former and no correlation with the latter (**i**). Binarized images were generated as above (two biological replicates from six PDXs were imaged). **j**, Correlative fluorescence microscopy, EM and NanoSIMS analysis after injection of BrDu, [^13^C_6_]glucose and [amide-^15^N]glutamine. More label is detected in WM^high^ compared to WM^low^. **k**,**l**, Cell-wise quantification of ^13^C/^12^C and ^15^N/^14^N ratios in WM^mix^ tumors. The data represent one biological replicate, two further are displayed in Extended Data Fig. 4i,j (*P* = 5.24 × 10^−17^ (**k**); *P* = 3.25 × 10^−5^ (**l**)). All box-and-whisker plots represent the following: line, median; box, IQR; whiskers, 1.5 × IQR limited by largest/smallest NEV. Significance was calculated with an unpaired two-tailed *t*-test. *P* values are represented by *<0.05, ** 0.001, ***<0.0001, ****<0.00001. Note that fluorescence images from WM^mix^ are depicted in both **a** and **c**, and **b** and **d** for better readability. Source data

**Extended Data Fig. 3:**
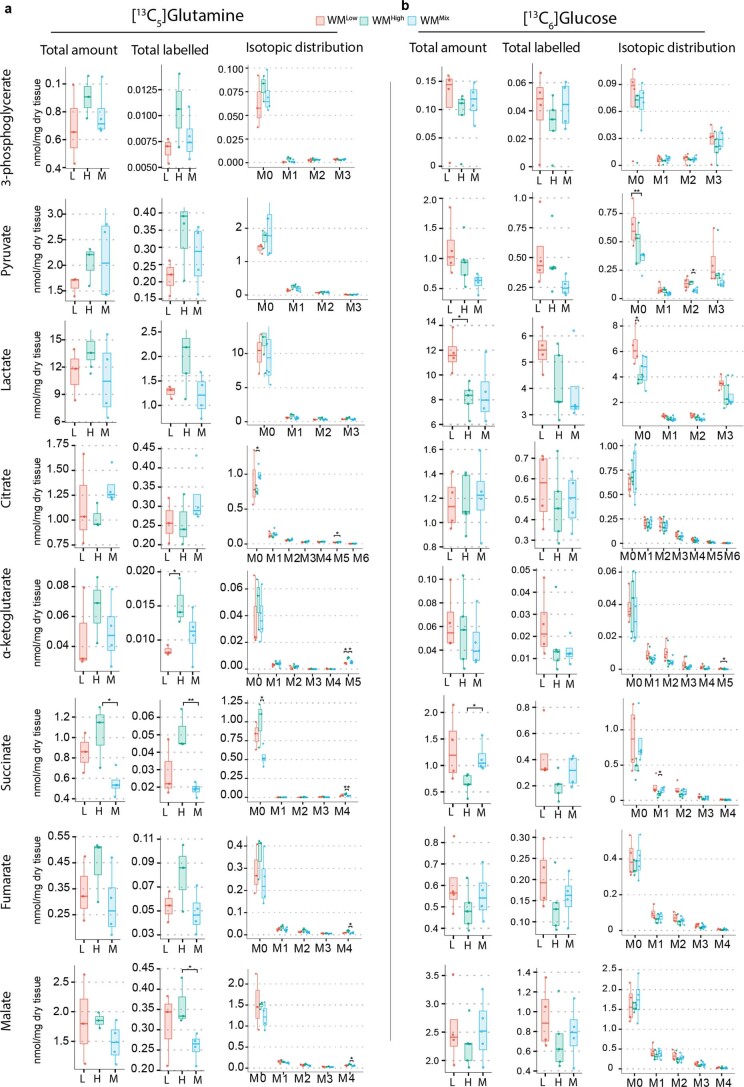
Activation of MYC leads to changes in carbon utilization. GC–MS analysis of WM tumors infused with [^13^C_5_]glutamine (**a**) or [^13^C_6_]glucose (**b**) shows an increase in glutaminolysis in WM^High^ and a trend towards more lactagenic metabolism in WM^Low^ tumors. Significance was calculated with an unpaired two-tailed t-test ([^13^C_5_]glutamine infusion WM^High^, n = 3; WM^Low^, n = 3; WM^Mix^, n = 4; [^13^C_6_]glucose infusions: WM^High^, n = 4 WM^Low^, n = 4, WM^Mix^, n = 4 tumors from independent animals). All box and whisker plots represent the following: Line: median, box: IQR, whiskers: 1.5x IQR limited by largest/smallest NEV. *P* value: *<0.05, **< 0.001. See also Fig. 3. Source data

**Extended Data Fig. 4:**
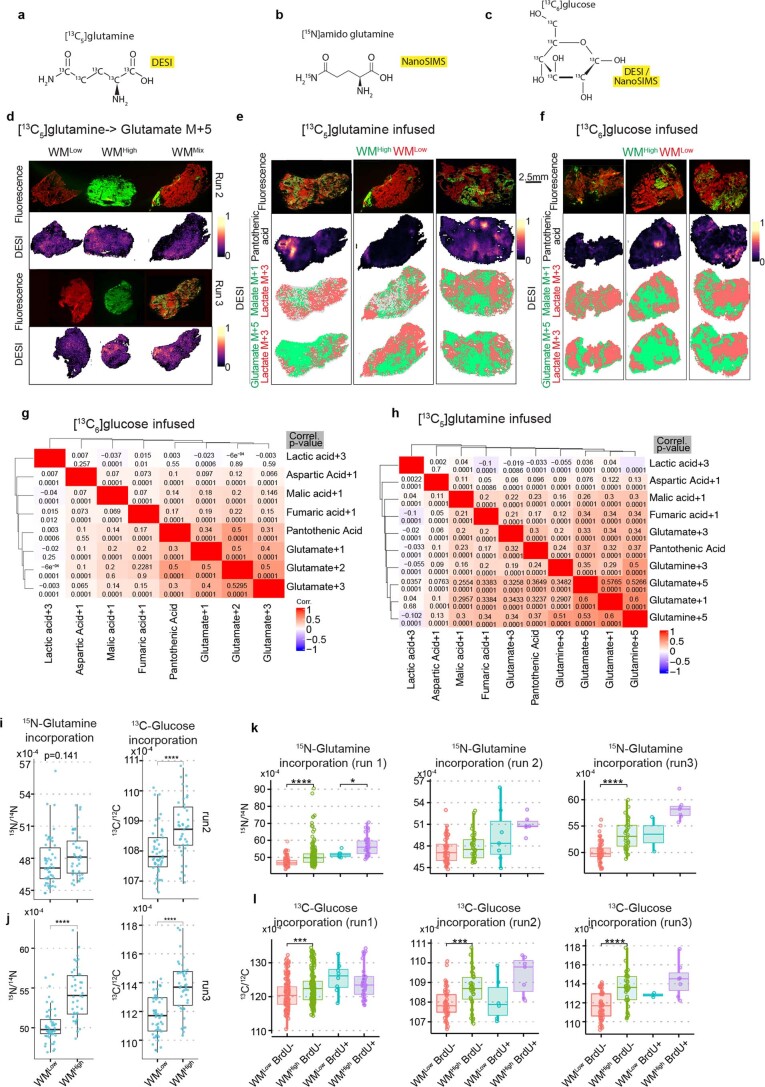
PA correlates with areas of high MYC and anticorrelates with lactagenic metabolism. **a-c**, Labeled compounds utilized in DEFFI or NanoSIMS imaging as indicated. **d**, Aligned post DEFFI fluorescent microscopy and glutamate M + 5 isotopologues measured by DEFFI after [^13^C_5_]glutamine infusion in two repeat runs (Run 1 is displayed in Fig. 3a). **e,f**, Post DEFFI fluorescent microscopy of WM^Mix^ tumors, shows increased PA in WM^High^ regions. The bottom panels are binarised representations of the labeled proxy compounds (lactate M + 3 isotopologue for lactagenic metabolism, malate M + 1 isotopologue for increased Krebs cycle, glutamate M + 5 for glutamine uptake) showing Krebs cycle activity corresponds with higher PA and WM^High^ areas. Ion intensities were corrected for natural abundance from individual metabolites and for each pixel the metabolite with the higher value after maximal intensity normalization was depicted. Note that for illustrative purposes some panels are also displayed in Fig. 3c,d. **g,h**, Table showing the pairwise correlation between different detected labeled compounds and PA. The top value represents the correlation and the bottom value the p value (n = 3 tumors from independent animals). **i**, **j**, Cell-wise quantification of ^13^C/^12^C and ^15^N/^14^N ratios in WM^Mix^ tumors (run 2 and run 3 each representing a tumor from an independent biological replicate, run 2: ^13^C/^12^C, p = 3.53e^−5^; ^15^N/^14^N, *P* = 0.141; run 3: ^13^C/^12^C, *P* = 3e^−8^; ^15^N/^14^N, *P* = 1.14e^−10^) **k**,**l**, Cell-wise quantification of ^13^C/^12^C and ^15^N/^14^N ratios in WM^Mix^ tumors stratified by BrDu incorporation status (n = 3 tumors from independent animals, ((k) run 1: Brdu^−^, *P* = 1.2e^−11^; BRDU^+^, *P* = 0.0312; run 2, *P* = 0.23; run 3, *P* = 5.94e^−9^; (l): run 1, *P* = 0.0001; run 2, *P* = 0.0004; run 3, *P* = 5.33e^−7^). All box and whisker plots represent the following: Line: median, box: IQR, whiskers: 1.5x IQR limited by largest/smallest NEV. Significance was calculated with an unpaired two-tailed t-test. *P* value: ****<0.00001. See also Fig. 3.

PA, as the precursor of CoA, has a central role in allowing carbons from glycolysis to enter the Krebs cycle, as well as for the Krebs cycle itself in the conversion of α-ketoglutarate to succinate. As glucose and glutamine are two of the main carbon sources contributing to this cycle^30,31^, we wanted to gain a better insight into the spatial distribution of their utilization in our tumor models. We thus infused WM tumors with either [^13^C_6_]glucose or [^13^C_5_]glutamine (Extended Data Fig. 4a,c) and traced their isotopically labeled downstream metabolites in situ through correlative DEFFI-MSI and fluorescence microscopy. As anticipated, glutamine catabolism in WM^high^ tumors was increased compared to their WM^low^ counterparts, as seen by higher levels of glutamate M+5 isotopologue in WM^high^ tumors following [^13^C_5_]glutamine infusion (Fig. 3a and Extended Data Fig. 4d). These results were largely in accordance with bulk primary tumor tissue analysis by gas chromatography (GC)–MS (Extended Data Fig. 3). Of note, the spatial component of MSI allowed us to reveal clear metabolic zonation within tumor tissues, where WM^high^ clones in the WM^mix^ tumors largely overlapped with areas of increased presence of [^13^C_5_]glutamine-derived glutamine and glutamate isotopologues as well as either [^13^C_6_]glucose- or [^13^C_5_]glutamine-derived Krebs cycle intermediates (Fig. 3a,b). These ^13^C-labeled isotopologues had a distribution pattern that was largely opposite to the localization of ^13^C-labeled lactate. While lactate can act as a systemic carbon carrier^32^, the individual cell producing it usually excretes it as a waste product. Increased lactate production can be triggered by hypoxia or a so-called Warburg-like metabolism and shunts carbon units away from the Krebs cycle and oxidative phosphorylation. We thus utilized isotopically labeled lactate as a proxy for diminished Krebs cycle activity and lactagenic metabolism and isotopically labeled malate as a proxy for increased Krebs cycle activity in [^13^C_6_]glucose- or [^13^C_5_]glutamine-infused tumors. By binarizing the ion images for the predominant proxy compound after maximal intensity normalization, a clear association of increased Krebs cycle activity with the WM^high^ clone was observed (Fig. 3c,d and Extended Data Fig. 4e,f). Pixel-wise correlations between different metabolites confirmed a close spatial association of Krebs cycle intermediates with PA, whereas lactate was poorly correlated with any of the compounds (Fig. 3e,f and Extended Data Fig. 4g,h). WM^high^ clones thus have higher levels of PA, which in turn correlates with a more active Krebs cycle.

To test whether this correlation was observed more widely, we administered [^13^C_5_]glutamine boluses to mice growing the aforementioned panel of breast cancer PDXs. While fewer labeled metabolites were detected overall, as this was a short-term bolus injection, we did see a positive correlation between glutamate M+5 and PA, which in turn correlates with MYC, but found no correlation of PA with labeled lactate (Figs. 2d and 3g–i and Extended Data Fig. 2f). Taken together, our results suggest that a MYC-driven increase in PA uptake supports an increased Krebs cycle activity.

As above, we sought to investigate whether increased glucose and glutamine uptake in WM^high^ areas was a cell-autonomous behavior by the WM^high^ clones. We thus adapted our ultra-high-resolution NanoSIMS-based correlative MSI protocol, by infusing WM^mix^ tumors with a mixture of [^13^C_6_]glucose and [amide-^15^N]glutamine (Extended Data Fig. 4b,c). Furthermore, we injected 5-bromodeoxyuridine (BrdU) 3 h before tumor collection, thus marking cells in S phase. This approach allowed us to image the two WM clones via fluorescence microscopy, while revealing cycling cells and tracing [^13^C_6_]glucose- and [amide-^15^N]glutamine-derived stable-isotope labels at subcellular resolution with the NanoSIMS (Fig. 3j). Of note, we saw strong ^15^N-labeling in nucleoli, an area of active ribosomal RNA production. This observation is consistent with the utilization of the amide nitrogen atom of glutamine in nucleotide biosynthesis. Conversely, glucose had a higher share of ^13^C-labeling detected in the cytosolic compartment, which is consistent with its role as a global carbon donor. Notably, WM^high^ cells overall had a significantly higher amount of label incorporation from both glucose and glutamine compared to WM^low^ cells, even in tightly intermingled tumor regions, arguing for a cell-autonomous effect of increased label uptake due to MYC activity (Fig. 3k,l and Extended Data Fig. 4i,j). This effect persisted, even when BrdU-positive cells, which have a particularly high amount of label incorporation due to the biosynthetic needs of S phase, were removed from the analysis, arguing for a MYC effect irrespective of the acute cell cycle status of the cells (Extended Data Fig. 4k,l). Overall, we conclude that heightened MYC activity increases PA uptake and metabolism, thus facilitating a more active Krebs cycle and metabolite uptake, in a cell-autonomous fashion.

## Depriving tumors of pantothenic acid reduces growth

**Fig. 4:**
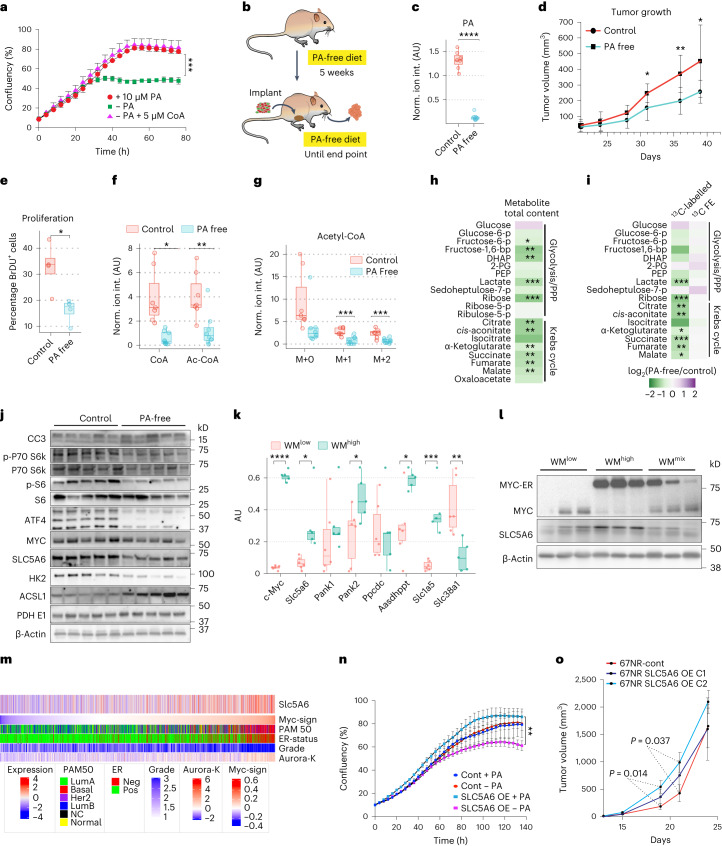
Tumors are dependent on pantothenic acid, whose import is regulated by MYC through SLC5A6 expression. **a**, IncuCyte analysis of cell growth of the high MYC 4T1 cells with and without PA (*n* = 3 technical replicates, error bars, mean ± s.e.m.; representative image of two biological replicates is shown, Holm–Sidak method *P* = 0.001248). **b**, Schematic of the experimental setup for diet alteration. **c**, LC–MS quantification of PA in extracts from HCI002 tumors grown with and without PA (*P* = 4.3 × 10^−10^). AU, arbitrary units. **d**, Growth of orthotopically transplanted HCI002 tumors grown with and without PA (**c**,**d**, control, *n* = 7; PA-free, *n* = 8 tumors from independent animals, mean ± s.d., *P* = 0.0284). **e**, Cell proliferation in tumors grown with and without PA quantified as BrdU-positive cells over total cells (control/PA-free, *n* = 4 tumors from independent animals, one section per tumor, *P* = 0.014). **f**,**g**, LC–MS analysis of HCI002 tumors grown with or without PA receiving a bolus of [^13^C_6_]glucose. CoA and acetyl-CoA (**f**) and labeled acetyl-CoA (**g**) (control, *n* = 7; PA-free, *n* = 8 tumors from independent animals; CoA, *P* = 0.00130; Ac-CoA, *P* = 0.0057; Ac-CoA M+0, *P* = 0.054; Ac-CoA M+1, *P* = 0.00080; Ac-CoA M+2, *P* = 0.0013). **h**, Total levels of selected metabolites from LC–MS analysis of HCI002 tumors grown with and without PA. **i**, Levels and fractional enrichment of ^13^C-labeled selected metabolites from LC–MS analysis of HCI002 tumors grown with and without PA receiving a bolus of [^13^C_6_]glucose. **j**, Western blot analysis of HCI002 tumors grown with and without PA. **k**, qRT–PCR of WM^high^ and WM^low^ tumors (WM^high^, *n* = 6; WM^low^, *n* = 5; WM^mix^, *n* = 6 tumors from independent animals, significant *P* values are 2.90 × 10^−11^, 0.013, 0.043, 0.010, 0.0005 and 0.006). **l** Western blot analysis of WM tumors. **m**, Stratification of tumors from the METABRIC dataset. **n**, IncuCyte growth analysis of the low MYC 67NR cells with ectopic expression of SLC5A6 with and without PA (*n* = 3 technical replicates; error bars, mean ± s.e.m.; representative image of three biological replicates is shown). **o**, Tumor growth of orthotopically transplanted 67NR cells with and without SLC5A6 over-expression (67NR control, *n* = 7; 67NR SLC5A6 OE C1/2, *n* = 6 tumors from independent animals; error bars, mean ± s.d.). Significance was calculated with an unpaired two-tailed *t*-test. All box-and-whisker plots represent the following: line, median; box, IQR; whiskers, 1.5 × IQR limited by largest/smallest NEV. *P* values indicated by *<0.05, ** 0.001, ***<0.0001, ****<0.00001. Source data

**Extended Data Fig. 5:**
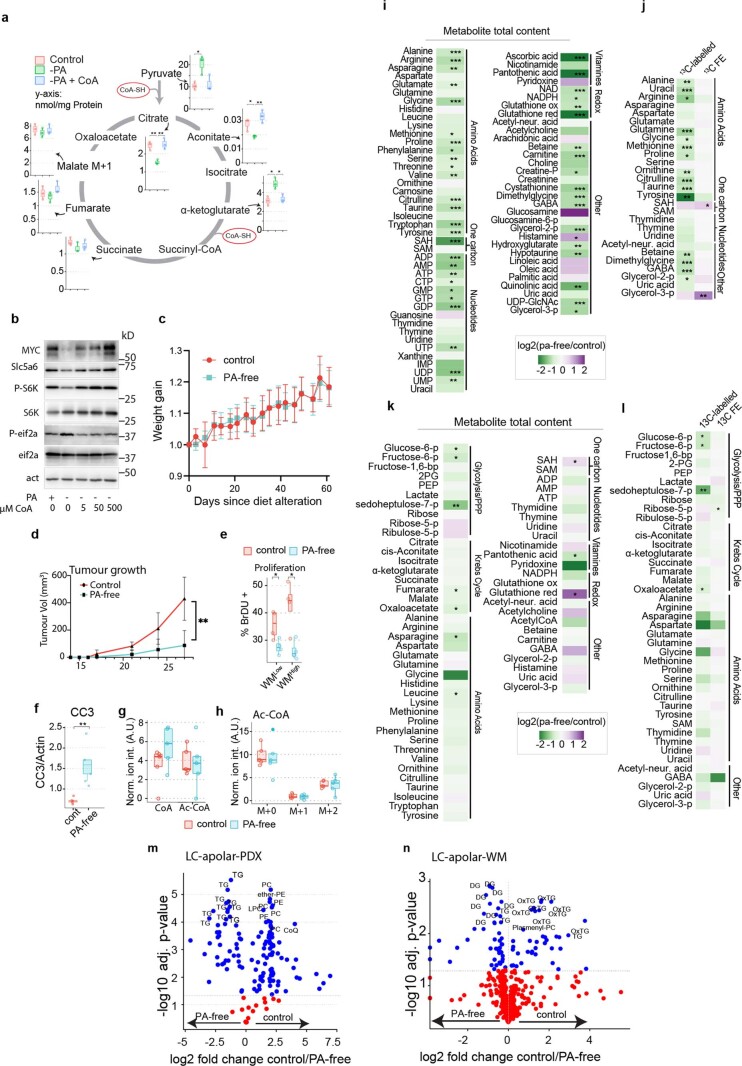
PA deprivation slows down mammary tumor growth in PDXs and WM tumors. **a**, Selected metabolites from GC^–^MS analysis of 4T1 cells with and without PA as well as CoA rescue. **b**, Western blot analysis of 4T1 cells with and without PA and with increasing amounts of CoA as a rescue (representative image of 3 biological repeats). **c**, Mouse weight during PA deprivation in the PDX-cohort (control diet, n = 7; PA-free diet, n = 8; error bar represents s.d.) **d**, Growth of WM^Mix^ tumors from mice fed a PA-free or control diet (n = 5 tumors from independent animals; error bar represent s.d., *P* = 0.02401). **e** Proliferation of WM^Mix^ tumors with and without PA quantified as BrDU-positive cells over total cells (n = 4 tumors from independent biological replicates, one section per tumor; WM^low^, *P* = 0.0304; WM^high^, *P* = 0.0126). **f**, Densitometric analysis of cleaved caspase 3 presence, normalised to actin in HCI002 tumors from mice fed PA-free or control diet (n = 5 tumor protein extracts from independent biological replicates, *P* = 0.00171). **g-h**, LC–MS analysis of WM^Mix^ tumors receiving a bolus of [^13^C_6_]glucose shows no significantly reduced amounts of free CoA-SH, Acetyl-CoA and labeled Acetyl-CoA isotopologues upon PA deprivation (n = 5 tumors from independent animals). **i**, Selected metabolites of LC–MS analysis of HCI002 tumors show widespread reduction in polar metabolite levels in tumors grown on PA-free diet. **j**, LC–MS analysis of HCI002 tumors receiving a bolus of [^13^C_6_]glucose shows widespread reduction in label incorporation in selected metabolites upon PA deprivation, but little difference in the fractional enrichment. **k**, LC–MS analysis of WM^Mix^ tumors shows widespread reduction in polar metabolite levels in tumors grown on PA-free diet. **l**, LC–MS analysis of WM^Mix^ tumors receiving a bolus of [13C_6_]glucose shows widespread reduction in label incorporation in selected metabolites upon PA deprivation, but little difference in the fractional enrichment. **m**, LC–MS analysis of the apolar fraction of HCI002 PDX tumors (n = 7 control, n = 8 PA-free) shows accumulation of triglycerides (TG), following PA deprivation (significance was calculated with a unpaired two-sided non-adjusted Student’s t-test). **n**, LC–MS analysis of the apolar fraction of WM^Mix^ tumors (n = 5 tumors from independent animals) shows accumulation of diglycerides (DG) and triglycerides (TG), following PA deprivation (significance was calculated with a unpaired two-sided non-adjusted Student’s t-test). All box and whisker plots represent the following: Line: median, box: IQR, whiskers: 1.5 x IQR limited by largest/smallest NEV. Significance was calculated with an unpaired two-tailed t-test. *P* value: *<0.05, ** 0.001. See also Fig. 4. Source data

The main downstream function of PA is mediated via CoA, although it is also used as a prosthetic group in fatty acid biosynthesis. To establish the requirement of PA and CoA for efficient tumor cell growth, we starved 4T1 mammary tumor cells of PA, which halted their proliferation and led to metabolite accumulation upstream of reactions involving CoA, as well as signs of cellular stress (Fig. 4a and Extended Data Fig. 5a,b). Notably, we could rescue this phenotype promptly, by supplementing the cells with CoA, highlighting the need for CoA as a downstream product of PA metabolism to sustain cell growth.

Given these observations, we wondered whether we could exploit therapeutically the reliance of tumor cells on this pathway. To reduce their systemic PA levels, mice bearing triple-negative breast cancer PDXs (HCI002, MYC high)^33^ or WM^mix^ tumors were fed a PA-free or control diet from 5 weeks before tumor implantation until tumor collection (Fig. 4b). The mice tolerated this treatment well (Extended Data Fig. 5c) and it significantly reduced tumoral PA levels (Fig. 4c). Notably, this coincided with a significant reduction in tumor growth and cell proliferation in both the HCI002 PDXs as well as in either clonal populations of the WM^mix^ tumors (Fig. 4d,e and Extended Data Fig. 5d,e). Note that under control diet conditions, WM^high^ clones in WM^mix^ tumors have a strong tendency toward increased proliferation, but this growth advantage is lost in the absence of PA (Extended Data Fig. 5e)^18^. Last, in the HCI002 tumors, PA deprivation also coincided with an increase in cell death (Fig. 4j and Extended Data Fig. 5f). Efficient tumor growth thus requires sufficient PA supply.

To understand how PA deprivation was affecting tumor metabolism, HCI002 tumors from PA-free diet or control diet-fed mice were analyzed via LC–MS after a [^13^C_6_]glucose infusion. As expected, in the case of the HCI002 PDXs on a PA-free diet, the amount of free CoA, as well as ^13^C-labeled acetyl-CoA, was significantly reduced (Fig. 4f,g). This was not the case in the WM^mix^ tumors (Extended Data Fig. 5g,h). This notwithstanding, in both tumor models the majority of glycolytic intermediates, Krebs cycle intermediates, both essential and non-essential amino acids and nucleotides were decreased when mice were fed a PA-free diet (Fig. 4h and Extended Data Fig. 5i,k), although, mirroring the CoA levels, this effect was more prominent in the PDXs compared to WM^mix^. Isotope tracing of [^13^C_6_]glucose revealed that total label incorporation into most compounds from central carbon metabolism was significantly reduced; however, ^13^C fractional enrichment did not change in the majority of metabolites, suggesting that tumor cells from mice on a PA-free diet do not compensate for their reduced capacity of glucose uptake and catabolism by metabolizing alternative compounds, but rather reduce the overall pool size of metabolites, retaining similar relative fluxes between glucose and other contributing carbon sources (Fig. 4i and Extended Data Fig. 5j,l). Comparing the apolar fraction of these tumors, revealed that the slower growing tumors propagated under PA-free conditions showed a larger pool of storage lipids such as diglycerides and triglycerides (Extended Data Fig. 5m,n).

Next, we sought to establish the adaptations in cellular signaling following PA deprivation. Of note, the PDX tumors displayed a slight reduction in c-MYC, but a robust reduction of the mTOR signaling pathway, an indicator for nutrient availability, as exemplified by reduced phosphorylation of its downstream targets p70S6K as well as S6K (Fig. 4j). This strongly implies that as a result of reduced PA in the diet, tumor cells signal a state of nutrient scarcity, which feeds back onto this central signaling axis. Consistent with reduced levels of many amino acids under PA-free conditions, ATF4, a known regulator of amino acid biosynthesis, was significantly reduced. ATF4 is a downstream target of mTOR signaling, the reduction of which might explain this observation (Fig. 4j)^34^. Consistent with a decreased glycolytic flux, the levels of the first enzyme of glycolysis, hexokinase (HK2), were reduced in PA-free tumors. Long-chain fatty acid CoA ligase, ACSL1, which is involved in both β-oxidation and fatty acid biosynthesis, behaved inversely and increased significantly under PA-free conditions, strengthening the notion that some of the carbon units from glucose are being diverted toward fatty acids. Notably, the expression of PDHE1, a component for the pyruvate dehydrogenase complex that shunts pyruvate into the Krebs cycle, was unchanged, indicating that flux regulation may precede this final step of glycolysis.

**Extended Data Fig. 6:**
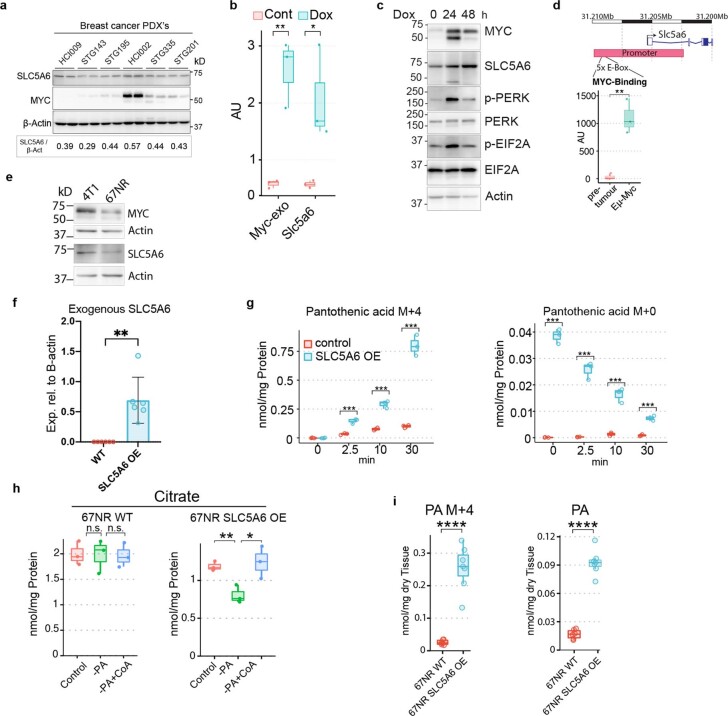
MYC upregulates SLC5A6 to increase PA uptake. **a**, Western blot analysis for MYC and SLC56A6 in human PDXs. **b**, qRT-PCR analysis of 67NR cells with Doxycycline-inducible MYC shows transcriptional upregulation of Slc5A6 upon MYC induction (n = 3 extracted RNA samples from independent biological replicates, error bar represents s.d., *P* = 0.0013). **c**, Western blot analysis of 67NR cells with Doxycycline-inducible MYC shows an upregulation of SLC5A6 upon MYC induction (representative image of 3 biological repeats). **d**, Publicly available *Myc* Chip-Seq data of pre-tumoral lymphocytes as well as overt lymphoma in an Eμ-Myc-driven model^72^ show invasion of an E-box cluster localized in the *Slc5A6* promoter. The data represents three biological replicates (error bar represents s.d., *P* = 0.0042). **e**, Western blot analysis of 4T1 and 67NR cells shows more MYC and SLC5A6 in 4T1 cells compared to 67NR cells. **f**, qPCR analysis of recombinant SLC5A6 expression (n = 5, error bar represents s.d.). **g**, GC–MS analysis of 67NR control and 67NR SLC5A6 over-expressing cells treated with stable-isotope labeled PA, shows an increased uptake of the labeled PA, and a higher baseline level of unlabeled PA in SLC5A6 over-expressing cells (n = 3 extracts from independent biological replicates, error bar represents s.d., PA M + 4, *P* = 1.5e^−5^, 0.0003, 4.30e^−6^; PA, *P* = 0.00032, 1.5e^−5^, 0.00038, 4.3e^−6^). **h**, GC–MS analysis of 67NR control and SLC5A6 over-expressing cells either starved for PA or rescued with CoA, shows no significant difference in the control cells between any of the treatments, while the SLC5A6 cells show a significant drop in citrate after PA deprivation that is readily rescued with CoA, implying metabolic reprogramming through SLC5A6 over-expression (n = 3 extracts from independent biological replicates, error bar represents s.d., control vs -PA, *P* = 0.0083; -PA vs -PA+CoA, *P* = 0.037). **i**, GC–MS analysis of stable-isotope labeled (M + 4) and endogenous PA in tumors grown from 67NR control cells or 67NR SLC5A6 over-expressing cells show an increased uptake of both endogenous and exogenous labeled PA (n = 6 control, n = 7 SLC5A6 OE extracts from tumors from independent biological replicates, error bar represents s.d., *P* = 0.0049, *P* = 0.0033). All box and whisker plots represent the following: Line: median, box: IQR, whiskers: 1.5x IQR limited by largest/smallest NEV. Significance was calculated with an unpaired two-tailed t-test. *P* value: *<0.05, ** 0.001. See also Fig. 4. Source data

We thus sought to gain mechanistic insights into the ability of Myc to enhance PA uptake in a cell-autonomous manner. PA is transported into cells alongside biotin and ɑ-lipoic acid by the multivitamin transporter SLC5A6 (also known as SMVT)^35^. We investigated the levels of SLC5A6 protein and messenger RNA in WM tumors and PDXs (protein only), where we saw a strong correlation between the transporter expression and MYC (Fig. 4k,l and Extended Data Fig. 6a). Consistent with increased PA catabolism, WM^high^ tumors have higher levels of pantothenic acid kinase 2 (PANK2) and aminoadipate-semialdehyde dehydrogenase-phosphopantetheinyl transferase (AASDHPPT), an enzyme that transfers a phosphopantetheine from CoA onto the acyl carrier domain of FASN (Fig. 4k). We confirmed that SLC5A6 expression is regulated cell-autonomously by MYC in a MYC-inducible cell line system, in which ectopic MYC expression is induced by doxycycline treatment in 67NR murine mammary gland tumor cells^18^. Ectopic MYC expression led to increased expression of SLC5A6 at both gene and protein levels (Extended Data Fig. 6b,c). Finally, the analyses of publicly available Chip-seq data^36^ revealed MYC binding to E-boxes in the *Slc5a6* promoter region (Extended Data Fig. 6d) confirming the direct transcriptional regulation of *Slc5a6* by MYC. Consistent with the connection between MYC and SLC5A6 observed in the preclinical models, we saw a clear correlation between higher grade, ER-negative and MYC signature-high tumors and the *SLC5A6* transporter (Fig. 4m) in the Molecular Taxonomy of Breast Cancer International Consortium (METABRIC) dataset of breast cancer samples^37^. These data prove a direct transcriptional activation of SLC5A6 by MYC.

Last, we set out to understand whether SLC5A6 was indeed responsible for cellular PA homeostasis and how this affected cellular metabolism and growth potential. The 4T1 cells and 67NR cells are sister cell lines from the same spontaneous murine breast cancer. The former has higher levels of both MYC and SLC5A6 compared to the latter (Extended Data Fig. 6e). We had previously noted that unlike the 4T1 cells under PA deprivation, the 67NR cells under the same condition did not promptly react with growth retardation and did not have profound changes in cellular metabolism (Fig. 4a,n and Extended Data Fig. 6h). We thus over-expressed SLC5A6 in these cells (Extended Data Fig. 6f). The baseline levels of PA were drastically increased, and the rapid uptake of a stable-isotope-labeled PA concomitant with a reduction of the unlabeled counterpart proved that this pool is highly dynamic (Extended Data Fig. 6g). SLC5A6 thus governs intracellular PA levels and can act as a bottleneck for PA import. Of note, SLC5A6 over-expressing cells had an increased proliferative capacity under PA replete conditions, but became dependent on PA, as its withdrawal inhibited their proliferation below the baseline of the control cells (Fig. 4n). Furthermore, their metabolism now responded to PA withdrawal similarly to the 4T1 cells (Extended Data Figs. 5a and 6h), arguing for a metabolic adaptation to high PA levels. To see whether these observations could be reproduced in vivo, we orthotopically transplanted two clones of the SLC5A6 over-expressing 67NR cells into Balb/c mice. Both clones initially grew faster, with one clone reaching statistical significance and the other one showing a strong trend (Fig. 4o), but this growth advantage was lost as the tumors reached around 1.5 cm^3^ in size. These results are consistent with the notion that at the onset of tumor growth, proliferation is the main driver in size gain, whereas later the establishment of a supportive microenvironment becomes more important. Indeed, all tumors showed widespread necrosis at the time of collection, arguing for insufficient support by the microenvironment. Last, we measured the levels of PA as well as the uptake of labeled [^13^C_3_,^15^N]PA in the tumors and saw a significant increase in both, showing that also in vivo SLC5A6 governs intracellular PA levels (Extended Data Fig. 6i).

## Discussion

**Extended Data Fig. 7:**
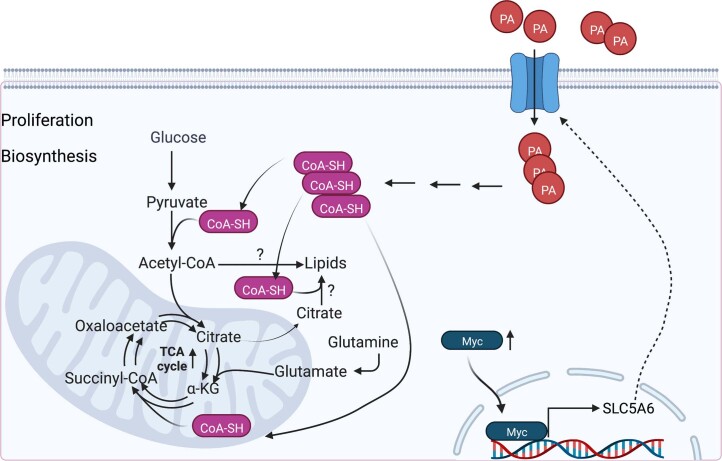
Model. MYC is upregulated in higher grade tumor areas. It can bind to the *SLC5A6* promoter and increase its transcription. This in turn increases PA intake, and enhances CoA synthesis, which facilitates a biosynthetic metabolism by enhancing Krebs Cycle activity, while shunting metabolites away from lactagenic metabolism. Whether fatty acid biosynthesis is also enhanced is not subject of this study, but consistent with our model (image created with BioRender.com).

In summary, we have shown that MYC increased PA levels within tumors through direct upregulation of the transporter SLC5A6 and that this is required to underpin the proliferative and biosynthetic programs of MYC (Extended Data Fig. 7). The need for such metabolic programs in tumors represents a tractable metabolic vulnerability.

Some historical data had noted the effect of PA on tumor growth^38^; our study, however, provides a functional link between high MYC expression, PA and downstream Krebs cycle activity. This is particularly pertinent, as vitamin supplementation is usually given to patients with cancer to counterbalance vitamin deficiencies caused by the side effects of chemotherapy on the gastrointestinal tract; however, our work as well as recent work from other groups^39^, show that under certain circumstances reducing vitamin availability to the tumor either by nutrient deprivation or possibly by blocking the cognate transporter might be advantageous. A very recent study has shown that a PI3K–Akt axis governs CoA biosynthesis from PA. Combined with our data, this represents an instance of metabolic oncogene cooperation, where MYC provides the cell with the ability to import the starting material (PA), whereas PI3K enhances downstream biosynthesis, in this case CoA^40^.

Of note, another recent study suggests that anti-tumorigenic T cells require high levels of CoA^41^. In our study we cannot account for T cell activity, as most of our models needed to be on an immunocompromised background to avoid rejection. T cells engage a very proliferative and biosynthetic metabolism once activated and are likely to engage a similar metabolic adaptation as the MYC-high tumor cells. It is thus an enticing thought that competition for the same nutrients might, in part, explain the lack of a therapeutic effect under immunotherapy. Consequently, being able to stratify based on known metabolic dependencies, and being able to predict antagonistic metabolic programs, will facilitate the development of bespoke therapies for a given tumor subtype. Last, we show how the utilization of in situ metabolomics and correlative imaging provide deeper insights into the local tumor biology, such as the finding of reduced lactagenesis in MYC-high tumor areas, which in turn helps to identify metabolic requirements driven by specific oncogenic profiles and their interplay with the microenvironment. Deploying this technology to other tumor systems will allow to extract more metabolic vulnerabilities that correlate with subclonal mutations or specific tumor subregions and thus lead to more targeted metabolic interventions.

## Methods

### Mice

#### Husbandry

All procedures and animal husbandry were carried out in accordance with the UK Home Office under the Animals (Scientific Procedures) Act 1986, and the Crick Animal Welfare and Ethical Review Body, which is delivered as part of the Biological Research Facility Strategic Oversight Committee (BRF-SOC), under project license P609116C5. Mice were caged in individually ventilated cages, on a 12-h light–dark cycle, at ambient temperature and a humidity of 55 ± 10%, with food and water ad libitum. The maximum tumor size permitted under the project license P609116C5 is 1.5 cm in diameter, which was never exceeded.

#### WM tumor generation

To generate spontaneous non-recombined tumors as a source of biclonal tumors, Rosa26-CAG-lox-STOP-lox-MYC-ERT2/ Rosa26-mTmG/MMTV-Wnt1 mice were used. The transgenes used to generate the cross were on the following backgrounds: MMTV-Wnt1, FVB/N; Rosa26-mTmG, C57BL/6J and Rosa26-CAG-lox-STOP-lox-MYC-ERT2:Balb/c. The tumors arose spontaneously between 4 and 8 months of age. Once palpable, tumors were desensitized to tamoxifen by daily intraperitoneal (i.p.) injection (100 µl, 10 mg ml^−1^ tamoxifen (Sigma) in olive oil with 10% ethanol). At 1.5 mm^3^ tumors were excised, cut into small fragments and cryopreserved in FBS (Gibco, A5256701, lot 2575507) with 10% dimethylsulfoxide (DMSO). To generate secondary tumors, fragments were surgically implanted into the number four fat pad of female NOD/Scid mice ((NOD.CB17-Prkdcscid/NCrCrl) at the age of 6–8 weeks. Once tumors were palpable, mice were treated as above. At 1.5 mm^3^ tumors were excised and digested in 5 ml additive-free DMEM with 1 mg ml^−1^ Collagenase/Dispase (Roche) for 1 h under shaking. Tumor cells were suspended in high-glucose DMEM (Thermo Fisher Scientific, 11960044) with 2 mM glutamine and 10% FBS (Gibco, A5256701, lot 2575507). To generate WM^high^ cells, Cre-expressing attenuated adenovirus (Ad5CMV-Cre, University of Iowa, VVC-U of Iowa-5) and Polybrene (8 μg ml^−1^), was added overnight. WM^low^ cells were not infected, but otherwise treated the same. Cells were washed extensively and eGFP- or tdTomato-positive cells were sorted with an Avalon sorter (BioRad). A total of 150,000 cells were then surgically implanted into the number four fat pad of 6–8-week-old NOD/Scid mice either as pure clonal populations or 1:1 mixtures. MYC-ER^T2^ was activated 3 d before collection by twice-daily i.p. injections of tamoxifen (100 µl, 10 mg ml^−1^ tamoxifen). On the day of collection, 3 h were left between the injection and further procedures, to have a fully active MYC-ER^T2^ construct. Tumors were collected at a size of 1.5 mm^3^. Mice were injected with BrdU (100 µl i.p., 10 mg ml^−1^ (Sigma)) 3 h before killing.

#### PDX tumor generation

The following previously described tumors were propagated via surgical fragment implant into 5-week-old female NSG mice subcutaneously^26^: HCI002, HCI009, STG143, STG195, STG335 and STG201; at the age of 5 weeks. Tumors took 2–10 months to form and were collected at a size of 1.5 mm^3^.

#### Diet modifications

A PA-free diet was formulated on the basis of a standard diet (composition is shown in Supplementary Table 2). PA absence was verified by LC–MS analysis. The control diet had the same composition as the PA-free diet, but with PA added. To avoid any interference with juvenile growth and puberty, diets were changed at 6 weeks of age. Diets were then introduced progressively over 4 d by increasing the amount of the PA-free/control diet and reducing the amounts of standard chow diet. In the meantime, tumor fragments were implanted into other Scid mice to grow WM tumors for in vitro recombination (WM only) and reimplantation or HCI002 tumors for reimplantation. Tumors were dissociated into a single-cell suspension and 750,000 (PDX) or 150,000 (WM tumors) cells were orthotopically implanted into the fourth inguinal fat pad of NOD/Scid mice. Mice were typically on a PA-free or control diet for 5 weeks before tumor implantation and remained on the diet until the tumors were collected. Dietary modification had no effect on mobility and responsiveness of animals nor their weight gain.

#### 67NR cell-derived tumor generation

The 67NR control and SLC5A6 over-expressing cells (150,000 cells in 80% Matrigel) were transplanted into female Balbc/CJ mice at the age of 7 weeks.

#### Stable-isotope labeling in vivo

The following compounds were utilized for stable-isotope labeling: [^13^C_6_]glucose, [^13^C_5_]glutamine, [amide-15N]glutamine and calcium pantothenate ([13C3,15N]β-alanyl), all from Goss Scientific. We either performed bolus injections of the compounds to study acute consumption or infusions, to measure the steady-state label incorporation or repeated boluses over many days to study label accumulation (PA ([^13^C_3_,^15^N]β-alanyl)). Mice were not fasted before stable-isotope administration and all experiments were performed in the early afternoon.

Boluses were performed as previously described^15^. Stable-isotope-labeled compounds were diluted in saline and injected intravenously (i.v.) into the tail vein of the mice. [^13^C_6_]glucose was administered in a single bolus of 0.4 mg g^−1^ body weight in approximately 100 μl final volume. [^13^C_5_]glutamine has limited solubility and was thus administered in two boluses 15 min apart for a total amount of 0.34 mg g^−1^ body weight. In both cases, 15 min after the last injection, tissues and blood were collected. Tumors and control tissues were swiftly excised and either snap-frozen in liquid nitrogen or fixed. Blood was extracted by cardiac puncture and serum was separated for metabolite analyses.

In the case of PA ([^13^C_3_,^15^N]β-alanyl), when used in mice to label WM tumors for NanoSIMS analysis 100-μl boluses of 3 mg ml^−1^ PA were administered during five consecutive days. Tumor collection was performed 5 h after the last administration. Conversely, in the mice transplanted with 67NR SLC over-expressing and control cells, a single bolus of PA ([^13^C_3_,^15^N]β-alanyl) at 0.086 mg g^−1^ body weight was administered 15 min before tumor collection.

Infusions of isotopically labeled nutrients were performed under general anesthesia (isoflurane) using an Aladdin AL-1000 pump (World Precision Instruments) following previously published protocols^42^. For [^13^C_6_]glucose, mice received a bolus of 0.4 mg g^−1^ body weight, followed by a 0.012 mg g^−1^ body weight per minute infusion for 3 h. For glutamine-derived stable-isotope infusions, mice received a bolus of 0.187 mg g^−1^ body weight, followed by a 0.005 mg g^−1^ body weight per min infusion for 3 h. For co-infusion of [^13^C_6_]glucose and [amide-^15^N]glutamine, a solution of 40 mg ml^−1^ glutamine and 96 mg ml^−1^ glucose was prepared and mice received a bolus of 0.187 mg of glutamine and 0.442 mg glucose per gram of body weight. This was followed by an infusion of 0.005 mg glutamine and 0.012 mg glucose per gram of body weight per minute during 3 h. At the end of the infusion, tissue and blood were obtained as described for the boluses.

Where indicated, mice were injected with BrdU (100 µl i.p., 10 mg ml^−1^ (Sigma)) 3 h before killing.

### Cell lines and culture conditions

The 4T1 and 67NR mouse mammary gland tumor cell lines were obtained from the Francis Crick Institute cell culture facility and were described previously. The 67NR–tet–cMYC–IRES–eGFP cells were described previously^18,43^.

The cell lines were authenticated using a standard protocol for identification of mouse cell lines using short tandem-repeat profiling. The profile is compared back to any available on commercial cell banks (such as ATCC and Cellosaurus). The species is confirmed by using a primer system, based on the Cytochrome C Oxidase Subunit 1 gene from mitochondria.

Cells were cultured in high-glucose DMEM (Thermo Fisher Scientific, 11960044) with 2 mM glutamine and 10% FBS (Gibco, A5256701, lot 2575507). Deviations are mentioned under the respective methods.

For in vitro PA uptake assays, cells were cultured in DMEM with 10 µM labeled PA ([^13^C_3_,^15^N]β-alanyl).

### Human breast biopsies

The institutional review board approved collecting all samples for this study at Imperial College Healthcare National Health Service Trust (Imperial College Healthcare Tissue Bank HTA license no. 12275 and Tissue Bank sub-collection no. SUR-ZT-14-043). The REC no. (REC Wales approval) is 17/WA/0161. All patients provided their consent to use their samples in this study. All methods were performed according to institutional and ethical guidelines. Patients undergoing surgery were recruited and 12 tissue samples were taken from a range of subtypes, which were identified in the histopathology assessment (Extended Data Table 2). Data were only obtained on patients who had consented to the utilization of tissue for research. Tumors had to be of a macroscopic size ≥2 cm to allow for adequate research tissue without compromising the clinical diagnosis. Where feasible, tissue was provided from the center of the tumor from non-necrotic areas. Upon collection, all tissue samples were stored at −80 °C.

### Metabolite extraction and analysis

#### Metabolite extraction

Snap-frozen tumors were ground with a liquid nitrogen-cooled mortar and pestle and subsequently lyophilized overnight in a FreeZone 4.5 Freeze Dry System (Labconco). Metabolites were extracted following published protocols^44^. Then, 5–10 mg of dried powder was weighed and extracted with 1.8 ml 2:1 chloroform:methanol. First, 0.6 ml methanol containing scyllo-inositol (10 nmol) and [^13^C_5_–^15^N]valine (5 µM final) as internal standards was added. This was followed by 1.2 ml chloroform containing margaric acid (C17:0, 10–20 μg). Samples were vortexed thoroughly and sonicated three times (8 min each) in a sonication bath at 4 °C. Samples were subsequently spun at 18,000*g* for 20 min and the supernatants were vacuum-dried in a rotational vacuum concentrator RVC 2–33 CD (Christ). Pellets were extracted with a methanol:water solution (2:1 v/v) as above and the two supernatants were combined and dried. Phase separation was performed with a 1:3:3 solution of chloroform:methanol:water. Polar (containing polar metabolites) and apolar (containing lipids) phases were stored separately at −80 °C until further processing. For the analysis of metabolites in blood, 5 μl serum was processed as above, without vortexing and sonication.

For in vitro assays, cells were plated in triplicate in six-well plates (250,000 cells per well) and cultured overnight before changing the medium to the indicated experimental conditions. At collection, cells were washed with PBS and snap-frozen. Subsequently, cells were scraped in 0.75 ml methanol, 0.25 ml chloroform and 0.25 ml water containing 1 nmol scyllo-inositol as an internal standard. Samples were vortexed, sonicated for 3 × 8-min bursts at 4 °C and incubated overnight at 4 °C. Samples were subsequently pelleted by centrifugation at 20,000*g* for 20 min at 4 °C. Phase separation was performed with a 1:3:3 solution of chloroform:methanol:water through the addition of 0.25 ml water to the supernatant. Polar and apolar phases were separated by centrifugation at 20,000*g* for 5 min at 4 °C. The polar phase was vacuum-dried in a rotational vacuum concentrator RVC 2–33 CD (Christ) for GC–MS analysis as detailed below.

#### GC–MS analysis

GC–MS analysis was performed following published protocols^15^. Part of the polar fraction was dried and washed twice with methanol. The first wash contained l-Nor-leucine (1.6 nmol per sample) as a run standard. For derivatization, metabolites were incubated with methoximation (Sigma, 20 µl, 20 mg ml^−1^ in pyridine) overnight followed by trimethylsilylation (20 µl N,O-bis(trimethylsilyl)trifluoroacetamide reagent (BSTFA) containing 1% trimethylchlorosilane (TMCS), Thermo Fisher). Samples were then analyzed using an Agilent 7890A-5975C GC–MS system. Splitless injection (injection temperature 270 °C) onto a 30 m + 10 m × 0.25 mm DB-5MS + DG column (Agilent J&W) was used, using helium as the carrier gas, in electron ionization mode. The initial oven temperature was 70 °C (2 min), followed by temperature gradients to 295 °C at 12.5 °C per min and then to 320 °C at 25 °C per min (held for 3 min). Under these conditions, glutamine and glutamic acid can spontaneously cyclize to pyroglutamic acid. The latter was thus used to assess glutamine levels in the tissue samples or plasma extracts, due to its preponderant abundance in blood. Metabolites were identified based on a mix of authentic standards, using the MassHunter software (Agilent, v.10.0.368). Label incorporation was calculated by subtracting the natural abundance of stable isotopes from the observed amounts^15,45^. Briefly, The level of enrichment of individual isotopologs (m+*x*) of metabolites was estimated as the percentage of the metabolite pool containing *x* ^13^C atoms after correction for natural abundance:

$${\rm{Enrichment}}\; {\rm{of}}\; m+{xx}=\frac{{\rm{Cm}}+x}{\varSigma {\rm{Cm}}+0+{\rm{Cm}}+1\ldots +{\rm{Cm}}+i}\times 100 \%$$

The percentage carbons enriched for a metabolite with *i* isotopologs was calculated by:

$$13{\rm{Cmet}}=\frac{m=1}{i}+2\frac{m+2}{i}\ldots +i\frac{m+1}{i}$$

#### LC–MS analysis

LC–MS analysis of the polar extracts was carried out following published protocols^46^. Samples were injected into a Dionex UltiMate LC system (Thermo Scientific) with a ZIC-pHILIC (150 mm × 4.6 mm, 5-μm particle) column (Merck Sequant). Solvent B was acetonitrile (Optima HPLC grade, Sigma-Aldrich) and solvent A was 20 mM ammonium carbonate in water (Optima HPLC grade, Sigma-Aldrich). A 15-min elution gradient of 80% solvent A to 20% solvent B was used, followed by a 5 min wash of 95:5 solvent A to solvent B and 5 min re-equilibration. Other parameters were a flow rate of 300 µl min^−1^; column temperature of 25 °C; injection volume of 10 µl; and autosampler temperature of 4 °C. MS was performed with positive/negative polarity switching using an Q Exactive Orbitrap (Thermo Scientific) with a HESI II probe. MS parameters were spray voltage of 3.5 kV and 3.2 kV for positive and negative modes, respectively; probe temperature of 320 °C; sheath and auxiliary gases were 30 and 5 AU, respectively; full scan range was 70 to 1050 m/*z* with settings of auto gain control (AGC) target and resolution as Balanced and High (3 × 10^6^ and 70,000), respectively. Data were recorded using Xcalibur software (Thermo Scientific), v.3.0.63. Mass calibration was performed for both ESI polarities before analysis using the standard Thermo Scientific Calmix solution. To enhance calibration stability, lock-mass correction was also applied to each analytical run using ubiquitous low-mass contaminants. Quality control samples were prepared by pooling equal volumes of each sample and analyzed throughout the run to provide a measurement of the stability and performance of the system. To confirm the identification of important features, some quality control samples were run in data-dependent top-N (ddMS2-top-N) mode, with acquisition parameters as follows: resolution of 17,500; AGC target under 2 × 10^5^; isolation window of m/z 0.4; and stepped collision energy 10, 20 and 30 in high-energy collisional dissociation mode. Qualitative and quantitative analysis was performed using Free Style v.1.5 and Tracefinder v.4.1 software (Thermo Scientific) according to the manufacturer’s workflows. For putative annotation, a CEU Mass Mediator tool was employed^47^.

LC–MS analysis of the apolar extracts was carried out following published protocols^48^. Lipids were separated by injecting 10-μl aliquots onto a 2.1 × 100 mm, 1.8-μm C18 Zorbax Eclipse plus column (Agilent) using a Dionex UltiMate 3000 LC system (Thermo Scientific). Solvent A was 10 mM ammonium formate in water (Optima HPLC grade, Fisher Chemical) and solvent B was water:acetonitrile:isopropanol, 5:20:75 (v/v/v) with 10 mM ammonium formate (Optima HPLC grade, Fisher Chemical). A 20-min elution gradient of 45% to 100% solvent B was used, followed by a 5 min wash of 100% solvent B and 3 min re-equilibration. Other parameters were a flow rate of 600 μl min^−1^; a column temperature of 60 °C; and an autosampler temperature of 10 °C. MS was performed with positive/negative polarity switching using a Q Exactive Orbitrap (Thermo Scientific) with a HESI II probe. MS parameters were a spray voltage of 3.5 kV and 2.5 kV for positive and negative modes, respectively; a probe temperature of 275 °C; sheath and auxiliary gases were 55 and 15 arbitrary units, respectively; full scan range was 150 to 2,000 m/z with settings of AGC target and resolution as Balanced and High (3 × 106 and 70,000), respectively. Data were recorded using Xcalibur software (Thermo Scientific), v.3.0.63. Mass calibration was performed for both ESI polarities before analysis using the standard Thermo Scientific Calmix solution. To enhance calibration stability, lock-mass correction was also applied to each analytical run using ubiquitous low-mass contaminants. To confirm the identification of significant features, pooled quality control samples were run in data-dependent top-N (ddMS2-top-N) mode, with acquisition parameters of mass resolution of 17,500; AGC target under 2 × 105; isolation window of m/z 0.4; and stepped collision energy of 10, 20 and 30 in high-energy collisional dissociation mode. Qualitative and quantitative analyses were performed using Free Style v.1.5 (Thermo Scientific), Progenesis QI v.2.4.6911.27652 (Nonlinear Dynamics) and LipidMatch v.2.02 (Innovative Omics)^49^. Radial plot representations were performed in R (v.3.6.2) using the R package volcano3D v.2.08 (ref. ^50^).

For the detection of CoA-SH and acetyl-CoA we used an Agilent 1200 LC system equipped with an Agilent Poroshell 120 EC-C18, 2.7 μm, 4.6 × 50 mm column was used. Then, 10 μl cleared samples were injected on the column. Mobile phase A was 40 mM ammonium formate at pH 6.8 and mobile phase B was LC–MS grade acetonitrile. Metabolites were separated at a flow rate 0.5 ml min^−1^, using the following elution conditions: 0–1 min, 2% B; 1–10 min, 2–98% B; and 10–12 min, 98% B. An Agilent accurate mass 6230 time-of-flight apparatus was employed. Dynamic mass axis calibration was achieved by continuous infusion of a reference mass solution using an isocratic pump connected to a dual Agilent Jet Stream ESI source, operated in the positive-ion mode. ESI capillary, nozzle and fragmentor voltages were set at 3,000 V, 2,000 V and 110 V, respectively. The nebulizer pressure was set at 40 psi and the nitrogen drying gas flow rate was set at 10 l min^−1^. The drying gas temperature was maintained at 200 °C. The sheath gas temperature and flow rate were 350 °C and 11 l min^−1^. The MS acquisition rate was 1.0 spectra s^−1^ and m/z data ranging from 50–1,200 were stored. The instrument routinely enabled accurate mass spectral measurements with an error of <5 ppm. Data were collected in the 2 GHz (extended dynamic range) mode and stored in centroid format.

### Western blot

Western blots were run using standard protocols for non-native protein detection. The following antibodies were used: MYC: AbCam, ab32072, 1:1,000 dilution; SLC5A6: Proteintech 26407-1-AP, 1:1,000 dilution; actin: Merck A3854 (HRP coupled), 1:25,000 dilution; cleaved caspase 3, Cell Signaling cat. no. 9664, 1:1,000 dilution; ATF4: AbCam, ab23760, 1:1,000 dilution; PDHE1: AbCam ab110330, 1:1,000 dilution; p70S6K: Cell Signaling, cat. no. 9202, 1:1,000 dilution; p-p70S6K: Cell Signaling, cat. no. 9234, 1:1,000; p-S6: Cell Signaling, cat. no. 4834s, 1:1,000 dilution; S6k: Cell Signaling, cat. no. 2217, 1:1,000 dilution; HK2: Cell Signaling, cat. no. 2867S, 1:1,000 dilution; AcSL1: Cell Signaling, cat. no. 4047, 1:1,000 dilution; p-PERK (Thr980): Cell Signaling, cat. no. 3179, 1:1,000 dilution; PERK: Cell Signaling, cat. no. 3192, 1:1,000 dilution; p-eIF2ɑ (Ser51): Cell Signaling, cat. no. 3398, 1:1,000 dilution; eIF2ɑ: Cell Signaling, cat. no. 5324, 1:1,000 dilution. The secondary antibodies were anti-rabbit-HRP, GE Healthcare, cat. no. NA934-1ML, 1:7,500 dilution; and anti-mouse-HRP, Invitrogen, cat. no. 62-6520, 1:7,500 dilution.

### RNA extraction and quantitative PCR

The 67NR-tet-cMYC cells were plated at 1.5 × 10^5^ cells in a six-well plate and cultured overnight before induction of MYC with doxycycline for 24 h. Cells were collected in TRIzol (Thermo Fisher). Similarly, 3 mg tumor tissue was homogenized in TRIzol. Total cellular RNA was extracted according to the manufacturer’s instructions. Subsequently, RNA was treated with DNase (Thermo Fisher, EN0525) and 1 µg RNA was incubated with 0.5 µM random primers. Complementary DNA was generated using Superscript III Reverse Transcriptase (Invitrogen, 18080044) and treatment with RNase OUT (Invitrogen, 10777019) according to the manufacturer’s instructions. Quantitative PCR reactions were performed in a ViiA 7 Real-Time PCR System (Thermo Fisher). PCR reactions were performed using Applied Biosystems Power SYBR Green PCR Master Mix (Thermo Fisher) in 10-µl reactions containing 5 pmol forward and reverse primer at 200 ng of cDNA. PCR conditions were an initial denaturation and polymerase activation for 10 min at 95 °C, followed by 40 cycles of 15 s and 95 °C and 60 s at 60 °C. These amplification cycles were followed by a melt-curve denaturation. Fold change and absolute abundance were determined using the 2^ΔΔCT^ and 2^ΔCT^ methods, respectively.

Primer sequences were designed using NCBI-PrimerBLAST:

*c-MYC*: (F) 5′-CCTTCTCTCCGTCCTCGGAT-3′, (R) 5′-TCTTGTTCCTCCTCAGAGTCG-3′; *Slc5a6*: (F) 5′-TTCACTGGCAACTGTCACGA-3′, (R) 5′-AGATACTGAGTGCTGCCTGG-3′; *Pank1*: (F) 5′-AAGAACAGGCCGCCATTCC-3′, (R) 5′-CTGCCGTGATATCCTTCGT-3′; *Pank2*: (F) 5′-TCACAGGCACCAGTCTTGGA-3′, (R) 5′-CCTGGCAAGCCAAACCTCT-3′;; *Ppcdc*: (F) 5′-CGTGCGTGTTGAGGTCATAG-3′, (R) 5′- GCCTGGGTCTAGAATCTGTCA-3′; *Aasdhppt*: (F) 5′-AAAGGGAAAGCCGGTTCTTG-3′, (R) 5′-ATTGAACCACGACCTGGAAA-3′; *Slc1a5*: (F) 5′-GCCTTCCGCTCTTTTGCTAC-3′, (R) 5′-GACGATAGCGAAGACCACCA-3′,

SLC5A6 exogenous: (F)TGGGCTGCTGTTACTTCCTG, (R) CACCCTCACGGTCTTGTTGA,

The sequence for primers for *Slc38a1* were obtained from Bian et al.^51^: (F) 5′-TACCAGAGCACAGGCGACATTC-3′, (R) 5′-ATGGCGGCACAGGTGGAACTTT-3′.

### Cloning

Using Gateway cloning (Invitrogen, Thermo Fisher), SLC5A6 (pDONR221_SLC5A6 was a gift from the RESOLUTE Consortium and G. Superti-Furga (Addgene plasmid #132194)) was inserted into the retroviral plasmid, pBabe-Puro (pBABE-Puro-gateway was a gift from M. Meyerson (Addgene plasmid #51070)).

### Retroviral gene transfer

Phoenix-AMPHO cells were seeded at 1.0 × 10^6^ cells in 6-cm plates and cultured overnight. Cells were subsequently transfected with 2 µg pBabe-SLC5A6 plasmid using PEI. After 16 h, the medium was replaced with 5 ml DMEM + 10% FBS and cells were cultured for an additional 48 h before collection of virus-containing supernatant. The supernatant was centrifuged at 1,000*g* for 5 min at 4 °C and filtered through a 0.45-µm syringe filter. Before transduction, 67NR cells were grown to 80% confluency and treated with 8 µg ml^−1^ Polybrene before infection with 0.5 ml virus-containing medium. After 24 h of transduction, cells were replated with 3 mg ml^−1^ puromycin for 72 h and maintained in the culture medium at 1 μg ml^−1^.

### Growth curves

Cells were washed with PBS before seeding at indicated confluency in high-glucose DMEM (custom made at the Francis Crick Institute) with 10% dialyzed FBS^52^ with or without 10 µM d-calcium pantothenate (Sigma, P5155) in 48-well plates. Cells cultured in the absence of pantothenate were cultured with or without Coenzyme A Trilithium Salt (EMD Millipore, 234101) at the indicated concentrations. Cells were imaged recurrently with the Incucyte S3 Live Cell Analysis System and confluency was measured using the Incucyte S3 Software (Sartorius) v.2021C.

### Sample preparation and DEFFI-MSI

Snap-frozen tumors were mounted onto cryosectioning chucks with a freezing drop of ice. The tumors were sectioned individually into 10-μm slices at −21 °C and thaw-mounted onto SuperFrost glass slides of 75 mm × 25 mm (Thermo Fisher Scientific). Sections were dried with a flow of nitrogen, placed in slide boxes and vacuum packed. They were stored at −80 °C until they were used for analysis. One set of PDXs was instead embedded in hydroxypropyl-methylcellulose (40–60 cP, 2% in H_2_O) and polyvinylpyrrolidone (average molecular weight 360,000) 7.5 and 2.5 g, respectively in 100 ml H_2_O and cryosectioned^53^. The downstream processing was as described above.

For human breast cancer biopsies each sample was cryosectioned into 10-μm thick parallel sections using a Cryostat Leica CM 1950 (Leica) set to −27 °C in the chamber and −25 °C in the sample holder. Tissue sections were put onto SuperFrost plus glass slides (Thermo Fisher Scientific). The slides were vacuum packed and stored at −80 °C until DEFFI-MSI analysis.

Imaging was carried out on the DESI imaging source (Prosolia) consisting of a two-dimensional sample holder moving stage coupled to a XEVO G2-XS QToF (Waters CorporationA), with the ion block temperature set at 150 °C. The DESI sprayer was converted to a DEFFI sprayer according to Wu et al. ^20^ by pulling the solvent capillary inwards^20^. A custom-built inlet capillary was heated up to 500 °C to assist the desolvation of secondary droplets. All images were acquired using HDImaging v.1.4 software in combination with MassLynx v.4.1 (Waters Corporation). Imaging parameters were set as follows: N_2_ gas pressure at 5 bar; 95:5 *v*/*v* methanol/water solvent was delivered by a nanoAcquity binary solvent manager (Waters Corporation) set at a flow rate of 1.5 µl min^−1^; sprayer voltage at 4.5 kV; sprayer angle of 75°; sprayer to surface distance of 2 mm; sprayer to inlet capillary distance of 1 mm; sprayer inlet capillary collection angle of 10°; emitter orifice distance of 150 µm; and orifice diameter of 200 µm. MS parameters were set as follows: *m*/*z* 50–1,000, one scan per second, horizontal acquisition speed at 100 μm s^−1^ with a lateral resolution of 100 μm, acquired in negative sensitivity MS mode. PA standard (100 ppm in 50:50 (*v*/*v*) methanol:water) was spiked onto tissue and left at room temperature for 15 min to dry. DEFFI-MS/MS imaging was then performed in negative sensitivity mode (precursor ion *m*/*z* of 218.10; collision energy of 12 v; one scan per second; and 100 µm × 100 µm pixel size). Once acquisition was finished, all tissue sections were stored at −80 °C before staining.

After DEFFI acquisitions, samples were rehydrated. For WM tumors, the slides were immediately cover-slipped and fluorescence was imaged on an Olympus VC120 slide scanner. PDX samples were also rehydrated and fixed for 10 min in 4% PFA. Staining was performed following standard IHC protocols.

#### DEFFI image analysis

DEFFI-MSI peaks used for the statistical modeling were extracted from the raw spectra. As a first step, peaks were centroided using the method described by Inglese et al.^22^. To include possible shoulder peaks, we selected those with a prominence greater than five. Peaks prominences were estimated using the ‘find_peaks’ function available in the Scipy package v.1.6.3 for Python^54^. A first intra-run peak matching was performed using MALDIquant v.1.19.3 (ref. ^55^) relaxed method with a search window of 50 ppm. This allowed SPUTNIK spatial filtering to remove signals that were detected outside the tissue or were associated with scattered images (number of eight-connected pixels <9) (ref. ^56^). Subsequently to the peak filtering, all non-tissue pixels were discarded. The filtered tissue peaks were mapped back to their original raw *m*/*z* values and mass recalibrated using the method described by Inglese et al.^22^. The recalibrated intra-run peaks were therefore matched using MALDIquant v.1.19.3 relaxed method. Matching of tissue peaks was performed with a search window of 50 ppm. If more than one peak was found in the search windows in the same spectrum, only the one with the highest intensity was retrieved. After this step, each run was assigned a set of common masses. To match the peak masses among different runs, for each DEFFI-MSI run *r*∈*R*, a representative list of peaks was calculated as the set of ordered pairs *X*_*r*_ = {(*m/z*_*p,r*_, *Y*_*p,r*_): *p*∈*P*_*r*_ }, where ${Y}_{p,r}={\sum }_{i=1}^{N}{y}_{i,p,r}/N$ is the arithmetic mean of the pixel-wise intensities *y*_*i,r*_ of the peak *p* with mass-to-charge ratio *m*/*z*_*p,r*_ and *P*_*r*_ is the set of common *m*/*z* values of the run *r*. The *m*/*z* values of the representative peaks lists of all *R* runs *X*′ = {*X*_1_, *X*_2_,…,*X*_*R*_} were matched together using the MALDIquant v.1.19.3 relaxed method with a tolerance of 20 ppm to generate a set *P*′ of inter-run common *m*/*z* values. Thus, this set of masses was common to all runs. The set of inter-run common *m*/*z* values was therefore filtered using a consensus approach. We removed all peaks *p*′∈*P*′ if the corresponding mean intensity *Y*_*p*′,*r*_ was equal to zero in at least one run *r*∈*R*. Final intensity matrices were median-scaling normalized (using median of non-zero intensities) and batch-effect corrected using ComBat (from SVA package v.3.34.0)^57^, with the batch equal to the acquisition run.

WGCNA v.1.70-3 (ref. ^58^) was employed to determine a consensus metabolic network following a similar approach described by Inglese et al.^21^. Only spectra corresponding to WM^high^ and WM^low^ tumors, not WM^mix^ were used to estimate the network. Signed hybrid^58^ adjacencies, corresponding to the only positive Pearson’s correlations between all pairs of features, were calculated from the individual runs, using a consensus soft power equal to the smallest value corresponding to an *R*^2^ > 0.85 across all runs. Tested values for the soft power ranged from 1 to 20. The adjacencies were subsequently normalized using the ‘single quantile’ method available in the WGCNA v.1.70-3 package for R, with the reference quantile set equal to 0.95 and combined into a consensus adjacency. Each element of the consensus adjacency matrix corresponded to the minimum adjacency value across the runs.

The consensus topological overlap matrix^59,60^, estimated from the consensus adjacency, was used to determine the network modules through hierarchical clustering (average linkage).

An initial set of clusters were determined using the dynamicTreeCut algorithm^61^, with the ‘hybrid’ algorithm and a smallest cluster size equal to 20. Module eigenmetabolites (MEs) were calculated from each module as the first principal-component scores of the merged runs spectra, using the only module features. The scores sign was reversed if Pearson’s correlation with the average image (calculated assigning to each pixel the mean intensity of their peaks) was negative. Modules with MEs correlated (Pearson’s correlation) more than 0.85 were considered identical and merged into a single module. Final MEs were recalculated from the merged modules. Association between modules and MYC was estimated using a linear regression model. First, for each module, the WM^high^ and WM^low^ values of the ME were binarized into ‘low-ME’ and ‘high-ME’ using the inter-run median value as threshold. Then, the proportion of ‘high-ME’ was calculated within each tissue section and used as the dependent variable of the regression model, whereas the binary tissue MYC condition of each pixel was used as independent variable. Because of its proportion nature, the dependent variable was modeled as a β-distributed (link = ‘logit’)^62,63^. ME models with a significantly different from zero slope (partial Wald test *P* < 0.05, Benjamini–Hochberg correction) were considered statistically associated to MYC. Finally, ME values for the WM^mix^ spectra were calculated projecting their module peak intensities on the corresponding loadings estimated from the WM^high^ and WM^low^ spectra.

All DEFFI single-ion images were maximal intensity normalized for individual ions within each run and scales were adjusted to values of 0–1 with the color palette adjusted with thresholding intensity at the 99.9th percentile to avoid artifacts caused by outliers, unless specified.

#### Dendrogram for ion colocalization of labeled metabolites

Mass-to-charge ratios (*m*/*z*) of selected metabolites were searched in the list of inter-run matched common *m*/*z* values. Theoretical *m*/*z* values were calculated from the deprotonated mass adding *k* times the δ *m*/*z* corresponding to the mass difference between ^13^C and ^12^C (ΔC = 1.003355 *m*/*z*). The value of *k* varied from zero to five, where *k* > 0 represented the isotopic forms. Features within an error of 10 ppm from the theoretical *m*/*z* value were considered as candidate matches. In the case of multiple candidates, the one corresponding to the highest mean intensity across all pixels was selected.

Matched features intensities corresponding to isotopes (*k* > 0) were adjusted for the natural isotopic abundance to estimate the intensity corresponding to the only labeled metabolites. The natural isotopic abundance was estimated from the observed raw monoisotopic intensities, with the probability of observing *k* ^13^C atoms, given *N* carbon atoms, modeled as binomially distributed, *Pr*(*X* = *k*) = Binom(*k*, *N*, *P*), where *P* = 0.01109 represents the probability of observing natural ^13^C isotopes. Pixel intensities corresponding to the labeled ^13^C component were therefore calculated subtracting the estimated natural ^13^C intensity from the raw intensity of the same pixel. Negative intensities could result from measurement errors. In these cases, we set the ^13^C intensity to zero, following the assumption that either no labeled molecules were detected or their abundance fell below the limit of detection of the instrument.

All three runs were included in the calculation. Intra-run Pearson’s correlations were estimated between the corrected intensities of the matched features. Subsequently, an inter-run consensus correlation matrix was calculated by taking the minimum values among the runs. Only WM^mix^ pixels were used for the calculation of the correlations.

The null hypothesis that consensus correlations between ion pairs is zero was tested using a permutation test. In each of 10,000 permutations, consensus correlations were calculated after shuffling the pixel intensities of the individual ion images. *P* values were calculated as $({\sum }_{i}{I}_{i}+1)/10,001$, where *I*_*i*_ is the indicator function, which is equal to 1 if the absolute permuted correlation is greater or equal than the original absolute correlation, 0 otherwise. *P* values of all pairwise correlations were adjusted using the Benjamini–Hochberg method.

A hierarchical clustering, with average linkage, was employed to partition the matched metabolites. One minus the consensus correlation was used as a distance measure. Clusters were identified by cutting the dendrogram at the level corresponding to two clusters.

### Automated segmentation and co-registration of PDX samples and human biopsies

Levels of MYC were identified by IHC (see below) in PDX samples and human biopsies. The IHC signal was deconvoluted using the inbuilt DAB staining deconvolution algorithm of the QuPath software package. The pixels devoid of any DAB staining from the deconvoluted IHC images were then removed by thresholding the green image channel at an intensity of 230. The remaining pixels were subsequently assigned as MYC positive. The image was then re-binned by a factor of 16 by summing the total number of MYC-positive pixels in any given 16 × 16 area using the MATLAB function ‘blockproc’ (Mathworks, image-processing toolbox). The resulting images (the MYC percentage proportions figures) were then clustered using the *k*-means clustering algorithm using Euclidean distance and *k* = 2 to differentiate regions that are high in MYC stain versus low. The binary image of the cluster that was highest in MYC signal versus those with low and no MYC was then extracted and registered to the MSI image. Areas of overt necrosis, staining and processing artifacts were excluded from the analysis by drawing masks. The masks were applied to the single-ion images for PA from MSI analysis as well as to the deconvoluted MYC staining before binarization, and within each tumor the average difference of mean levels of PA between regions of high and low MYC was assessed by a linear mixed-effects model fitted with the ‘glmmTMB‘ v.1.1.4 package for R (random intercepts were considered for run and tissue ID). We then plotted the observed proportions connected by a line per sample, with a black dot representing the predicted mean of the two groups. The error bars represent the confidence interval of the predictions for the two groups (Supplementary Fig. 1).

### Immunohistochemistry/immunofluorescence

Immunofluorescence staining was performed against BrDU, eGFP and Tomato using the following antibodies. BrDU (BD, 347580, 1:100 dilution); eGFP *(Abcam, ab6683, 1:100 dilution); and RFP (Rockland, 600-401-379, 1:500 dilution). Secondary antibodies were Alexa Fluor-647 donkey anti-mouse, A-31571, Alexa Fluor-555 donkey anti-rabbit, A-31572, Alexa Fluor-488 donkey anti-goat, A-11055, all at 1:500 dilution. Slides were blocked for 10 min in 4% PFA at room temperature and rehydrated in PBS–Tween (0.1%) for 10 min. Slides were then transferred into a citric buffer (10 mM at pH 6.0) and boiled in a microwave for 15 min. Slides were cooled under running tap water and blocking was performed in PBS–Tween (0.1%) with 3% BSA for 1 h at room temperature. Primary antibodies were incubated overnight, whereas secondary antibodies were incubated for 1 h at room temperature. Nuclei were stained with Hoechst (0.1 μg ml^−1^, Sigma). To assess proliferation in WM tumors, three visual fields per sample were segmented by clones (WM^high^ and WM^low^) and total nuclei as well as BrdU-positive nuclei were quantified using the cell counting plugin in ImageJ. To assess proliferations in PA-deprived and control HCI002 PDXs, BrdU staining was carried out as above, slides were scanned with an Olympus VC120 slide scanner and nuclei as well as BrdU-positive cells were quantified on the whole slide using the Qpath cell-counting feature. IHC staining for c-MYC was carried out on frozen sections post-DEFFI for PDX samples and on the consecutive slice for human biopsies. The secondary antibody was a biotinylated goat anti-rabbit (BA-1000, Vector, 1:250 dilution) for 45 min at room temperature. The ABC kit (PK-6100 from Vector) was incubated for 30 min and DAB for 10 min.

### NanoSIMS

#### Electron microscopy embedding

For NanoSIMS analysis, WM^mix^ tumors were grown as described above. At 3 h before collection, BrdU (100 μl, 10 mg ml^−1^) was administered to the mice i.p. Either boluses of stable-isotope labeled calcium pantothenate ([^13^C_3_,^15^N]β-alanyl) or a co-infusion of [^13^C_6_]glucose and [amide-^15^N]glutamine were administered to the mice as described above. Once removed tumors were fixed overnight in freshly prepared 4% paraformaldehyde in 0.1 M phosphate buffer (PB) at pH 7.4 and stored at 4 °C. Following initial fixation, samples were embedded in 2% low-melting-point agarose (A4018-50G, Sigma-Aldrich) in 0.1 M PB and 150-μm sections were collected using a vibrating knife ultramicrotome (VT1200S, Leica), using a speed of 1 mm s^−1^ and an amplitude of 0.75 mm. Excess agarose was removed from the sections and tissue was stored in a 24-well plate in 0.1 M PB at 4 °C.

Sections were then transferred onto a glass slide, cover-slipped in PB and the whole section imaged in a single plain with a confocal microscope (Leica, Falcon SP7) at ×100 magnification (objective HCPL APO CS2 ×10, NA 0.4). Regions of interest (ROIs) were chosen and a z-stack of approximately 70-μm depth at ×100 magnification was imaged over an area of approximately 1.5 mm^2^.

Imaged sections were then removed from the glass slide and embedded using a protocol adapted from the NCMIR method^64^. Sections were post-fixed in 4% paraformaldehyde/2.5% glutaraldehyde in 0.1 M PB at pH 7.4 for 1 h at room temperature. Samples were then washed in 0.1 M PB (5 × 3 min) before being post-fixed in 2% reduced osmium (2% osmium tetroxide/1.5% potassium ferricyanide) at 4 °C for 1 h. Sections were washed (5 × 3 min in dH_2_O), stained in 1% thiocarbohydrazide for 20 min at room temperature, washed again (5 × 3 min in dH_2_O) and stained in 2% osmium tetroxide for 30 min at room temperature. Finally, samples were washed (5 × 3 min in dH_2_O) before being left overnight in 1% uranyl acetate at 4 °C. The following day sections were washed (5 × 3 min in dH_2_O) and then stained en bloc with lead aspartate (pH 5.5) for 30 min at 60 °C. After a final wash (5 × 3 min in dH_2_O), sections were dehydrated using a graded series of alcohol (20%, 50%, 75%, 90%, 100% × 2, 20 min each) followed by infiltration with Durcupan resin (44610-1EA, Sigma-Aldrich) (1:1 resin:ethanol overnight and 100% resin for 24 h). Sections were then flat embedded using Aclar (L4458, Agar Scientific) and polymerized at 60 °C for 48 h.

#### Targeted single-section large area montaging

Polymerized sections were removed from the Aclar and blocks were prepared from the ROI. The ROI was identified by aligning the overview ×100 confocal image of the whole section to an overview image of the now-embedded section, acquired using a stereo microscope (MC205C stereo, DMC 4500 Camera, Leica Microsystems) in Photoshop (Adobe). The identified area was then removed from the resin with a small bit of excess on each side using a razor blade and the excised block was mounted on a metal pin (10-006002-50, Labtech) using silver epoxy (604057, CW2400 adhesive, Farnell), which was polymerized at 60 °C for 1 h^65^. The sample was removed from the oven and any excess resin and silver epoxy was trimmed using a glass knife on an ultramicrotome (EM UC7, Leica Microsystems). The resin block was shaped into a square with one corner removed so that it was asymmetric, to aid in orientation of the block in the serial block-face (SBF) scanning electron microscope (SEM). Finally, the block face was trimmed until the tissue was reached. The block was then sputter coated with 10 nm platinum (Q150S, Quorum Technologies) and loaded into a 3View2XP (Gatan) attached to a Sigma VP SEM (Zeiss) with focal charge compensation (Zeiss). The SBF SEM was used as a ‘smart trimming’ tool, allowing visual assessment of the tissue structure during cutting until the EM images of the block face, collected using a BSE detector (3View detector, Gatan), matched with the structures imaged in the ×100 confocal *z*-stack. When the *z*-plane containing the ROI had been reached, the sample was removed from the SBF SEM, the block was re-trimmed using a glass knife to a sub-area of the ROI approximately 400 μm × 200 μm and 200-nm sections were cut from the block face using an ultramicrotome (EM UC7, Leica Microsystems) and a 6-mm histo diamond knife (DiATOME). Sections were collected onto silicon wafers (G3390, Agar Scientific) using an eyelash and dried on a hotplate at 70 °C for 10 min. The silicon wafers were then mounted onto SEM stubs (10-002012-100, Labtech Electron Microscopy) using adhesive carbon tabs (15-000412, Labtech Electron Microscopy) and loaded into a Quanta FEG 250 SEM (Thermo Fisher Scientific). Tiled images of the whole section were collected using Maps software (v.1.1.8.603, Thermo Fisher Scientific) using a low-voltage high-contrast backscattered electron detector (vCD, Thermo Fisher Scientific). Images were collected using a voltage of 2.5 kV, a spot size of 3, a dwell time of 5 ms, a working distance of 6 mm and a pixel resolution of 10 nm. Individual images from the tiled sequence were exported to TIFF files and aligned into a single image using the TrackEM2 plugin in Fiji^66^. Both the exported single image and the corresponding resin section on a silicon wafer were then sent to NanoSIMS for targeted analysis, where they were loaded into the NanoSIMS (Cameca NanoSIMS 50L, Cameca/Ametek). Before analysis, the pulse height distributions of the electron multiplier detectors were analyzed and their voltage gains and thresholds adjusted if necessary. They were then further examined by measuring the C^−^ and CN^−^ count rate on adjacent detectors used to measure the ^13^C/^12^C and ^12^C^15^N/^12^C^14^N isotope ratios. This step is imperative to measure accurate isotope ratios. The NanoSIMS 50L has seven detectors which were then moved to the appropriate radii in the magnetic sector with fixed magnetic field to measure the following masses: ^12^C, ^13^C, ^12^C^14^N, ^12^C^15^N, ^31^P, ^79^Br and ^81^Br. Images were typically acquired at 50 μm × 50 μm field width or 25 μm × 25 μm with a 300-μm D1 aperture (D1-2) to provide a mosaic correlating with regions previously analyzed by fluorescence and scanning EM. Nono-SIMS images were acquired using Cameca NanoSIMS N550L software v.4.4. and were processed and quantitative data extracted using the OpenMIMS plugin for ImageJ.

The acquisition modes were overlaid using the BigWarp plugin in Fiji. For better visualization, the pseudocolor look-up-table (LUT) was changed in the [^13^C_6_]glucose and [^15^N]amido-glutamine co-infused samples.

### MYC signature in PDXs

Based on the microarray expression data from the PDXs used^26,67^ we downloaded the c6 oncogenic signatures from the Molecular Signatures Database^68,69^ and applied the GSVA package, v.1.48.3 (ref. ^70^) to infer sample specific MYC pathway activation. As the PDX cohort might not represent all types of breast cancers, we included the 1,980 METABRIC samples from elswehere^37^ and quantile-normalized them together before converting the expression values into *z* scores. For pathways that had a subset of genes that should be upregulated and another subset that should be downregulated, the final score was obtained as upregulated − downregulated. We compared the scores with the log-intensity expression with a scatter-plot.

### Metabolic pathway analysis

Global feature extraction was carried out using Progenesis QI (Nonlinear Dynamics) tool following parameters used for the LC–MS analysis method (FWHM, 70,000, minimum chromatographic peak width of 0.166 min, min. intensity threshold of 10^5^). Features with coefficient of variation below <30% across replicates from at least one class of the WM^high^, WM^low^ or WM^mix^ tumor samples were further used for untargeted metabolic pathway analysis using MetaboAnalyst v.5.0 (ref. ^71^). A feature table consisting of 1,562 input *m*/*z* features from polar and apolar sample analysis of both polarities were processed using the mummichog algorithm. A feature significance cutoff of 0.05 *P* value for a Student’s *t*-test between WM^high^ and WM^low^ was used and enriched pathways were identified using *Homo* *sapiens* (KEGG) pathway library (mass accuracy threshold of 10 ppm). For calculation of the enrichment score for the identified pathway, the number of significant feature hits from individual pathways with fold change difference higher in either WM^high^ or WM^low^ samples was estimated. The enrichment score is then calculated with a modified mummichog algorithm by the significant hits coming from individual sample types rather than all significant hits and by using Fisher’s exact test *P* value for pathway enrichment to scale the enrichment score. Enriched pathways that consist of pathway size >1 in the library were retained for the analysis.

### Conditional probability for ion coexpression with WM^high^ clones

The list of metabolites associated with enriched pathways in WM^high^ tumors were compared to the DEFFI-MS data for deprotonated ions. Conditional probabilities *P* (metabolite level | myc level) were estimated from the multi-run merged normalized and batch-effect-corrected DEFFI data. Metabolite intensities were discretized into low, mid and high levels by thresholding to the *t*_1_ = 33th and *t*_2_ = 66th percentiles (*y* ≤ *t*_1_⇒low, *t*_1_ < *y* ≤ *t*_*2*_⇒mid, *y* > *t*_*2*_⇒high). The conditional probabilities were calculated as a fraction of pixels with either low, mid or high intensity, stratified by MYC level. Only pixels from WM^low^ and WM^high^ were used for the estimation. The calculated conditional probabilities were represented as a heat map using R v.3.6.2 (ref. ^72^) and the ComplexHeatmap v.2.2.0 (ref. ^73^) package.

### METABRIC dataset analysis

Transcript levels of SLC5A6 were evaluated in human breast cancer samples from the publicly available METABRIC microarray gene expression dataset^37^. Samples were subsequently annotated with molecular subtype based on Prediction Analysis of Microarray 50 allocations^74^, estrogen receptor status, grade, size, number of lymph nodes positive and proliferation (expression of Aurora Kinase A). Thereafter, samples were ordered according to MYC signature expression (average transcript levels of 335 genes), retrieved from a previously published core MYC expression signature (consisting of 398 genes)^75^. Data analyses were undertaken using R v.3.6.1 (ref. ^72^), the ComplexHeatmap v.2.2.0 (ref. ^73^) and Circlize v.0.4.15 (ref. ^76^) packages.

### Chip-seq

Publicly available data from Sabo et al.^36^ were downloaded from the Gene Expression Omnibus repository under accession no. GSE51011. An E-box rich region inside the murine *Slc5a6* promoter was identified using the genomic sequence extracted from the Ensembl database and the respective region was identified in the Chip-seq raw data. The respective binding intensities of MYC to this region were plotted for pre-tumor as well as tumor cells.

### Statistics

Pairwise comparisons were generally carried out using the Student’s *t*-test in Excel or using the R package ggplot2 v.3.4.0 and ggpubr v.0.4.0. No statistical methods were used to predetermine sample sizes but our sample sizes are similar to those reported in previous publications^15,18^.

When possible, mice from the same litter were randomized into groups. To avoid litter effects, different cell types (for example WM^high^, WM^low^ and WM^mix^) were implanted into mice from different litters in a random fashion. This was also the case in all studies with diet alteration. Before changing the diet, litters were mixed and randomized. Samples were randomized for metabolomics analysis. In preclinical model experiments, tumor size and mouse weight measurements were blinded as well as treatment regimes. DESI/DEFFI imaging was carried out blind and agnostic to the underlying tumor genotypes. Data analyses were not performed blind to the conditions of the experiments. Data were only excluded due to technical failure (for example over confluence, at start of an experiment, bad melting curve in qRT–PCR). No data were systematically excluded. Only mice with failed tumor grafting or tumor-unrelated health issues were excluded. Mice with failed metabolite administration due to improper cannulation and mice with failed tumor grafting were excluded.

For the NanoSIMS study with calcium pantothenate (dual label ^13^C and ^15^N), the ^13^C trace was recorded, but not analyzed, as it failed to show a signal above background, due to a higher natural abundance of ^13^C compared to ^15^N.

Statistical methods embedded in R-code-based image analysis, such as the ion colocalization analysis and the correlation dendrogram, are indicated in the respective sections.

All box-and-whisker plots represent the following: line, median; box, IQR; whiskers, 1.5 × IQR limited by largest/smallest NEV.

Data distribution was assumed to be normal but this was not formally tested.

### Reporting summary

Further information on research design is available in the Nature Portfolio Reporting Summary linked to this article.

## Supplementary information

Supplementary Information Supplementary Figs. 1, 2 and 3 and Supplementary Table 2.

Reporting Summary

Supplementary Table 1 List of ions constituting metabolic modules identified in WM tumors.

## Source data

Source Data Fig. 1 LC–MS analysis data of polar and apolar metabolites in WM tumors.

Source Data Fig. 2 LC–MS analysis of CoA levels in WM tumors and quantification of single-cell PA-derived ^15^N/^14^N ratio detected by NanoSIMS in WM tumors.

Source Data Fig. 3 Quantification of single-cell ^13^C/^12^C and ^15^N/^14^N ratios detected by NanoSIMS in WM tumors.

Source Data Fig. 4 LC–MS analysis of polar metabolite in HCI002 tumors grown with and without PA. LC–MS analysis of CoA and acetyl-CoA in HCI002 tumors grown with and without PA. Analysis of the indicated gene expressions in WM^high^ and WM^low^ tumors. HCI002 tumor growth with and without PA. Growth of tumors from 67NR cells with and without ectopic expression of SLC5A6.

Source Data Fig. 4 Uncut western blots for Fig. 4j,l.

Source Data Extended Data Fig. 2 GC–MS data of PA level analysis in PDXs. Quantification of subcellular ^15^N/^14^N ratios detected by NanoSIMS in WM tumors. Quantification of single-cell PA-derived ^15^N/^14^N ratio detected by NanoSIMS in WM tumors (second biological replicate).

Source Data Extended Data Fig. 3 GC–MS analysis of polar metabolites from WM tumors labeled with ^13^C-glutamine. Values are nmol of metabolite per mg dry tissue. GC–MS analysis of polar metabolites from WM tumors labeled with ^13^C-glucose. Values are nmol of metabolite per mg dry tissue.

Source Data Extended Data Fig. 5 GC–MS analysis of extracts from 4T1 cells grown either with or without PA as well as rescued with CoA. Values are area under the curve. LC analysis of CoA and acetyl-CoA in WM tumors grown with and without PA. LC–MS analysis of polar metabolite levels in WM tumors grown with and without PA. LC–MS analysis of apolar metabolite levels in HCI002 tumors grown with and without PA. LC–MS analysis of apolar metabolite levels in WM tumors grown with and without PA. Growth of WM tumors in the presence and absence of PA. Quantification of BrdU signal in WM tumors grown in the presence and absence of PA.

Source Data Extended Data Fig. 6 GC–MS analysis of polar metabolites in 67NR cells with SLC5A6 over-expression. GC–MS analysis of the effect of PA deprivation on polar metabolites in 67NR cells. GC–MS analysis of the effect of PA deprivation on polar metabolites in 67NR cells with SLC5A6 over-expression. GC–MS analysis of polar metabolites in tumors from 67NR cells with and without the ectopic expression of SLC5A6. Expression of genes upon the induction of ectopic MYC expression in 67NR cells.

Source Data Extended Data Fig. 6 Uncut western blots for Extended Data Fig. 6a,c,e.

## Figures and Tables

**Extended Data Table 1:**
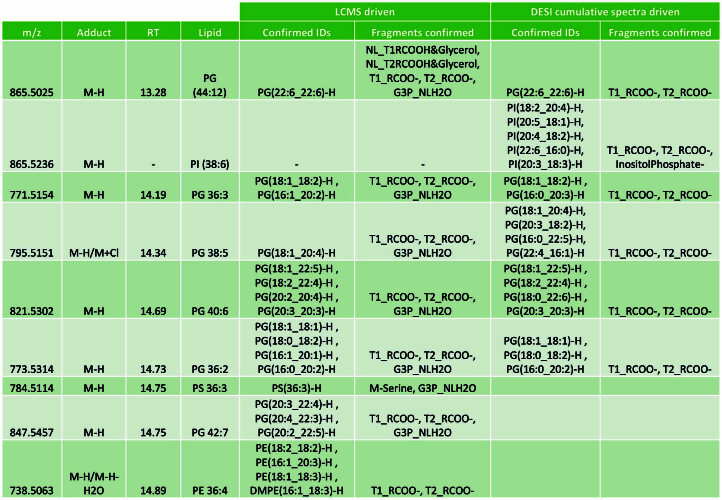
List of ions confirmed with MS/MS following LC–MS and/or DEFFI-MSI pipeline

**Extended Data Table 2:**
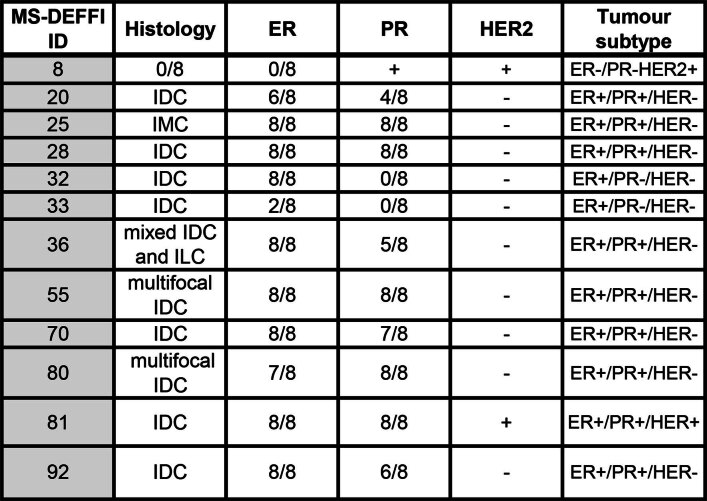
List of human breast cancer biopsies

## Data Availability

For Chip-seq analysis, publicly available data from Sabo et al. were downloaded from the Gene Expression Omnibus repository under accession no. GSE51011 (ref. ^36^). The datasets generated during and/or analyzed during the present study, including MSI data, IHC data for PDX samples and human biopsy samples and raw GC–MS data for WM tumors are available at 10.25418/crick.23925252. Source data are provided with this paper.

## Code availability

All codes used to carry out the data analysis in the current study including the analysis of MSI data, Myc pathway activation and IHC data clustering are available at 10.25418/crick.23925426.
