# Supplementary material for: Vitamin B5 supports MYC oncogenic metabolism and tumor progression in breast cancer
Source: Nat Metab. 2023 Nov 9;5(11):1870–86. doi: 10.1038/s42255-023-00915-7 (PMC10663155; doi:10.1038/s42255-023-00915-7)
Supplement: Supplementary file 1 — Supplementary Figs. 1, 2 and 3 and Supplementary Table 2. [file 42255_2023_915_MOESM1_ESM.pdf]

nature metabolism

Letter

https://doi.org/10.1038/s42255-023-00915-7

# Vitamin $B_5$ supports MYC oncogenic metabolism and tumor progression in breast cancer

In the format provided by the authors and unedited

**Supplementary Table 2. PA control diet (TestDiet 57W5)**

**I N G R E D I E N T S (%)**

| | |
|---|---|
| Corn Starch | 39.7486 |
| Casein - Vitamin Tested | 20.0000 |
| Maltodextrin | 13.2000 |
| Sucrose | 10.0000 |
| Soybean Oil | 7.0000 |
| Powdered Cellulose | 5.0000 |
| AIN 93G Mineral Mix | 3.5000 |
| AIN 93 Vitamin Mix | 1.0000 |
| L-Cystine | 0.3000 |
| Choline Bitartrate | 0.2500 |
| t-Butylhydroquinone | 0.0014 |

**NUTRITIONAL PROFILE**

| | |
|---|---|
| ***Protein, %*** | ***18.3*** |
| Arginine, % | 0.70 |
| Histidine, % | 0.52 |
| Isoleucine, % | 0.96 |
| Leucine, % | 1.73 |
| Lysine, % | 1.45 |
| Methionine, % | 0.52 |
| Cystine, % | 0.37 |
| Phenylalanine, % | 0.96 |
| Tyrosine, % | 1.01 |
| Threonine, % | 0.77 |
| Tryptophan, % | 0.22 |
| Valine, % | 1.14 |
| Alanine, % | 0.55 |
| Aspartic Acid, % | 1.29 |
| Glutamic Acid, % | 4.08 |
| Glycine, % | 0.39 |
| Proline, % | 2.36 |
| Serine, % | 1.10 |
| Taurine, % | 0.00 |
| ***Fat, %*** | ***7.1*** |
| Cholesterol, ppm | 0.00 |
| Linoleic Acid, % | 3.58 |
| Linolenic Acid, % | 0.55 |
| Arachidonic Acid, % | 0.00 |
| Omega-3 Fatty Acids, % | 0.55 |
| Total Saturated Fatty A | 1.05 |
| Total Monounsaturated Fatty Acids, % | 1.54 |
| Polyunsaturated Fatty Acids, % | 3.78 |
| ***Fiber (max), %*** | ***5.0*** |
| ***Carbohydrates, %*** | ***63.2*** |
| ***Minerals*** | |
| Calcium, % | 0.51 |
| Phosphorus, % | 0.32 |
| Potassium, % | 0.36 |
| Magnesium, % | 0.05 |
| Sodium, % | 0.13 |
| Chloride, % | 0.22 |
| Fluorine, ppm | 1.0 |
| Iron, ppm | 40 |
| Zinc, ppm | 35 |
| Manganese, ppm | 11 |
| Copper, ppm | 6.0 |
| Cobalt, ppm | 0.0 |

| | |
|---|---|
| Iodine, ppm | 0.21 |
| Chromium (added), ppm | 1.0 |
| Molybdenum, ppm | 0.14 |
| Selenium, ppm | 0.24 |

### *Vitamins*

| | |
|---|---|
| Vitamin A, IU/g | 4.0 |
| Vitamin D-3 (added), IU/g | 1.0 |
| Vitamin E, IU/kg | 81.6 |
| Vitamin K, ppm | 0.75 |
| Thiamin, ppm | 4.8 |
| Riboflavin, ppm | 6.7 |
| Niacin, ppm | 30 |
| Pantothenic Acid, ppm | 16 |
| Folic Acid, ppm | 2.1 |
| Pyridoxine, ppm | 5.8 |
| Biotin, ppm | 0.2 |
| Vitamin B-12, mcg/kg | 28 |
| Choline Chloride, ppm | 1,250 |
| Ascorbic Acid, ppm | 0.0 |

## Flow Chart for automated k-means based tissue segmentation

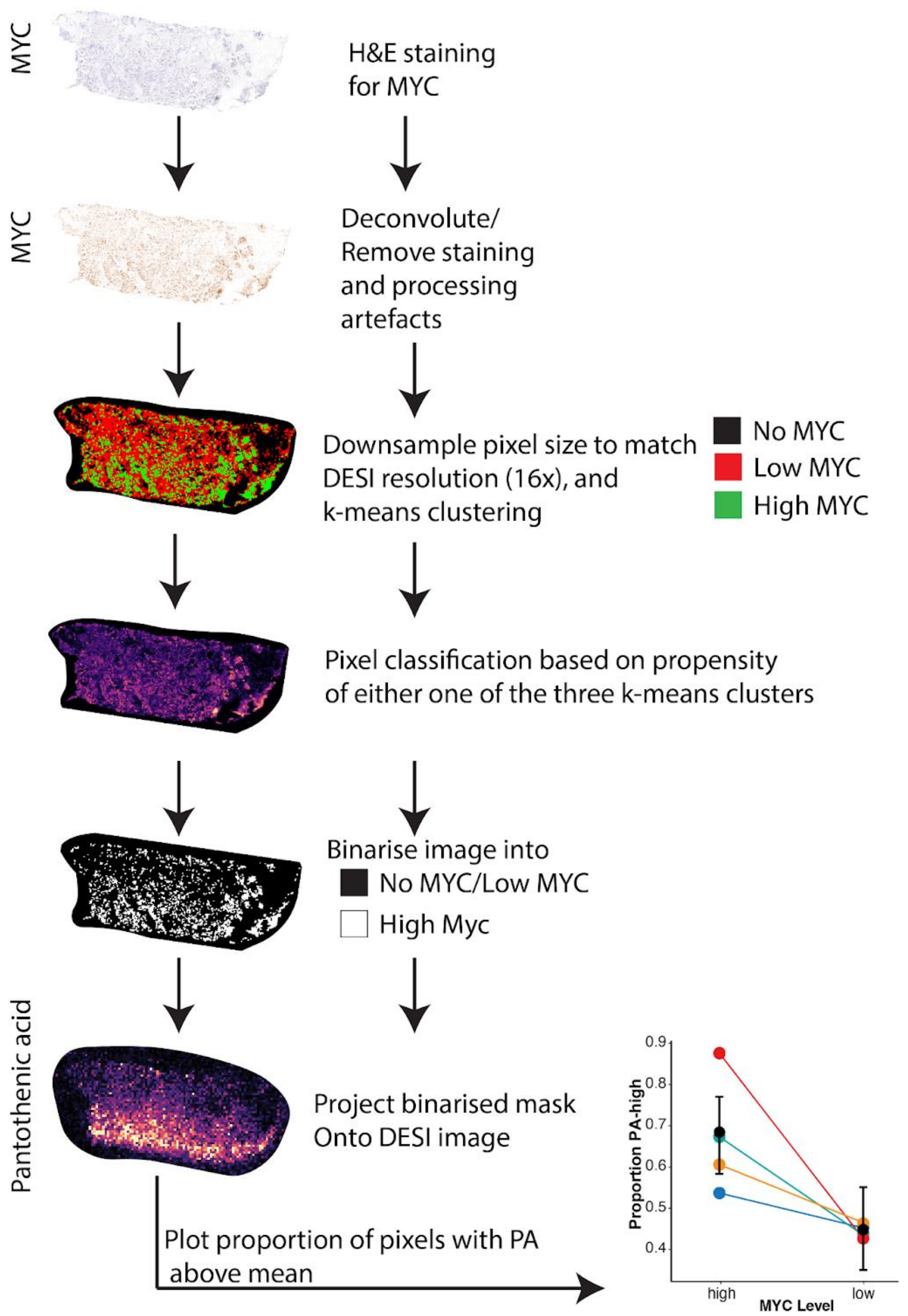

**Supplementary Figure 1. An automated k means-based tissue segmentation.**

Levels of MYC were identified by Immunohistochemistry in PDX samples and human biopsies after removal of areas containing staining artefacts. These areas were also excluded from downstream analysis. IHC signal was deconvoluted using the inbuilt DAB staining deconvolution algorithm of the QuPath software package. The pixels void of any DAB staining from the deconvoluted IHC images were then removed by thresholding the green image channel at an intensity of 230. The remaining pixels were subsequently assigned as MYC positive. The image was then re-binned by a factor of 16 by summing the total number of MYC positive pixels in any given 16x16 area using the Matlab function “blockproc” (Mathworks, Image processing toolbox). The resulting images (the MYC percentage proportions figures) were then clustered using the k-means clustering algorithm using Euclidean distance and k = 2 to differentiate regions that are high in MYC stain vs. low. The binary image of the cluster that was highest in MYC signal vs those with low and no MYC was then extracted and registered to the MSI image. Within each tumour the average difference of mean levels of PA between regions of high and low MYC was assessed by a linear mixed effect model fitted with the `glmmTMB` package for R (random intercepts were considered for run and tissue ID).

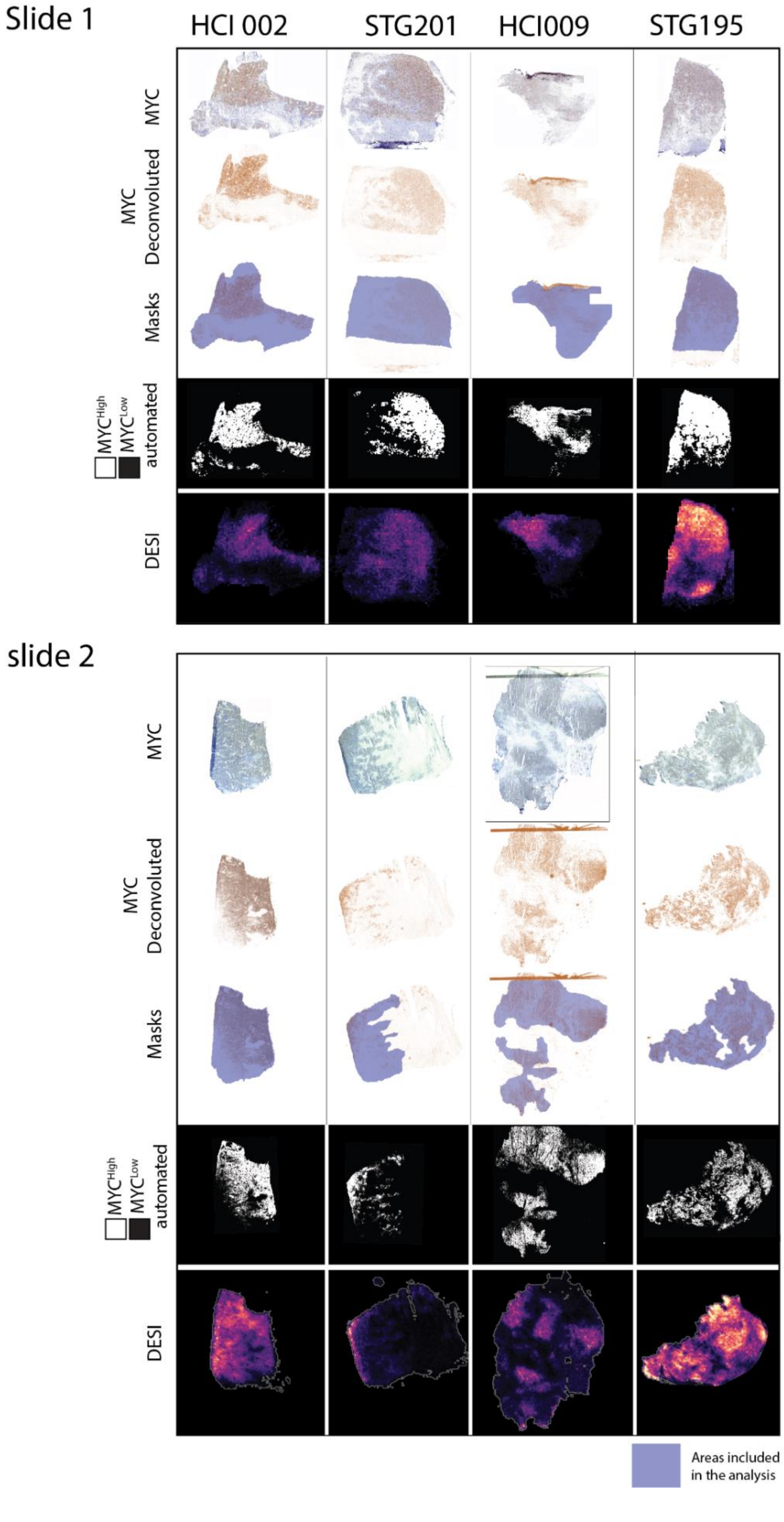

Slide 1
HCI 002
STG201
HCI009
STG195
MYC
MYC Deconvoluted
Masks
MYC^High
MYC^Low
automated
DESI
slide 2
MYC
MYC Deconvoluted
Masks
MYC^High
MYC^Low
automated
DESI
Areas included in the analysis

**Supplementary Figure 2. Outputs of automated segmentation of MYC staining in PDX samples.**

Levels of MYC were identified by immunohistochemistry on the DEFFI-imaged section. IHC signal was deconvoluted using the inbuilt DAB staining deconvolution algorithm of the QuPath software package. Areas of overt necrosis and staining artefacts were excluded from the analysis by drawing masks over processing artefacts. The masks were also applied to the single ion images for PA from MSI and within each tumour the average difference of mean levels of PA between regions of high and low MYC was assessed as described above.

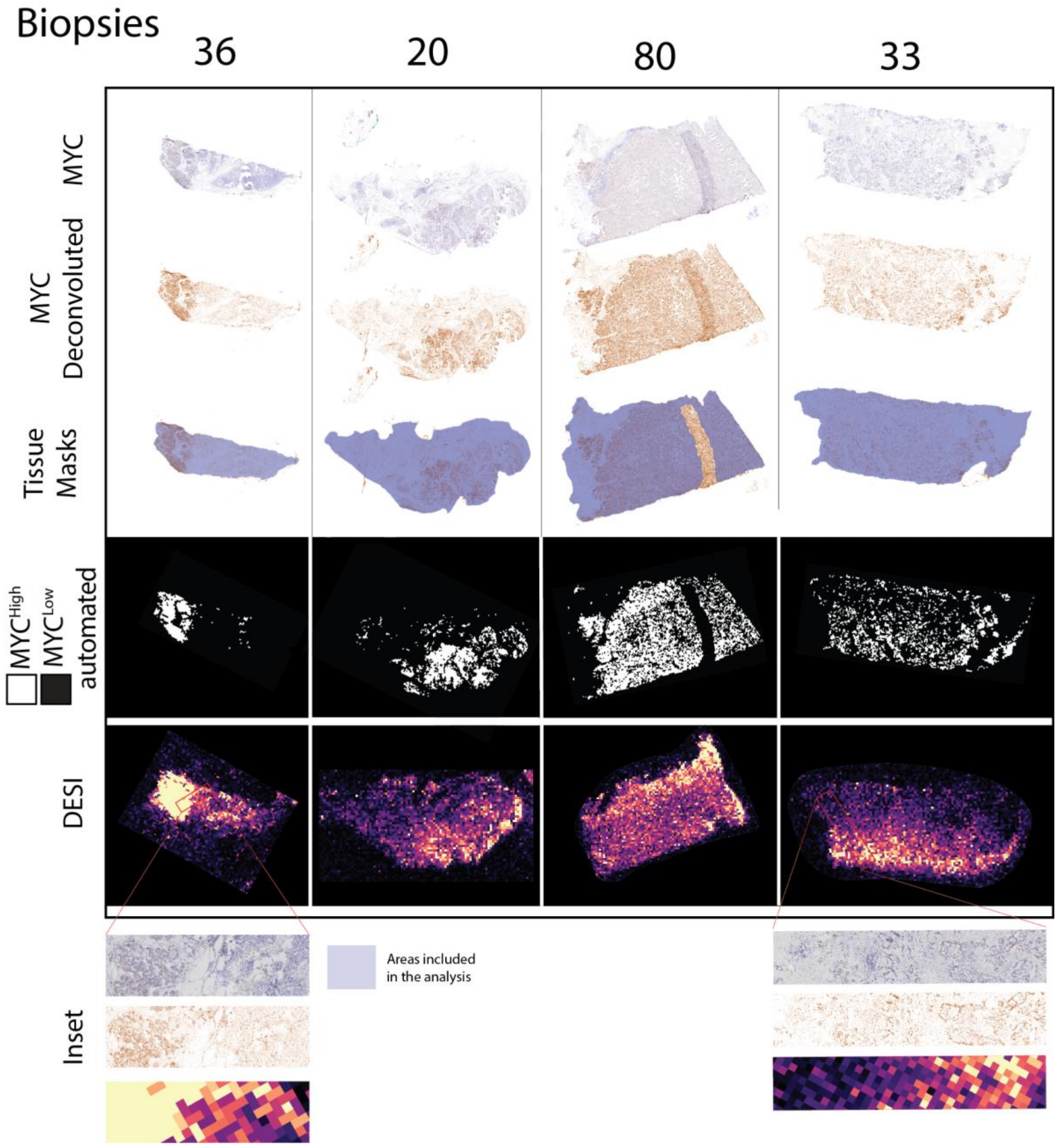


**Supplementary Figure 3. Outputs of automated segmentation of MYC staining in human breast cancer biopsies.**

Levels of MYC were identified by immunohistochemistry on the section consecutive to the DEFFI-imaged section. IHC signal was deconvoluted using the inbuilt DAB staining deconvolution algorithm of the QuPath software package. Areas of overt necrosis and staining artefacts were excluded from the analysis by drawing masks over processing artefacts. The masks were also applied to the single ion images for PA from MSI and within each tumour the

average difference of mean levels of PA between regions of high and low MYC was assessed as described above.
