# Supplementary material for: Vitamin B5 supports MYC oncogenic metabolism and tumor progression in breast cancer
Source: Nat Metab. 2023 Nov 9;5(11):1870–86. doi: 10.1038/s42255-023-00915-7 (PMC10663155; doi:10.1038/s42255-023-00915-7)
Supplement: Supplementary file 2 — Reporting Summary [file 42255_2023_915_MOESM2_ESM.pdf]

nature portfolio

Corresponding author(s): Mariia Yuneva, Peter Kreuzaler

Last updated by author(s): Mar 14, 2023

Reporting Summary

Nature Portfolio wishes to improve the reproducibility of the work that we publish. This form provides structure for consistency and transparency in reporting. For further information on Nature Portfolio policies, see our Editorial Policies and the Editorial Policy Checklist.

Statistics

For all statistical analyses, confirm that the following items are present in the figure legend, table legend, main text, or Methods section.

n/a	Confirmed	
☐	☒	The exact sample size (*n*) for each experimental group/condition, given as a discrete number and unit of measurement
☐	☒	A statement on whether measurements were taken from distinct samples or whether the same sample was measured repeatedly
☐	☒	The statistical test(s) used AND whether they are one- or two-sided<br>*Only common tests should be described solely by name; describe more complex techniques in the Methods section.*
☒	☐	A description of all covariates tested
☒	☐	A description of any assumptions or corrections, such as tests of normality and adjustment for multiple comparisons
☐	☒	A full description of the statistical parameters including central tendency (e.g. means) or other basic estimates (e.g. regression coefficient) AND variation (e.g. standard deviation) or associated estimates of uncertainty (e.g. confidence intervals)
☐	☒	For null hypothesis testing, the test statistic (e.g. *F*, *t*, *r*) with confidence intervals, effect sizes, degrees of freedom and *P* value noted<br>*Give P values as exact values whenever suitable.*
☒	☐	For Bayesian analysis, information on the choice of priors and Markov chain Monte Carlo settings
☒	☐	For hierarchical and complex designs, identification of the appropriate level for tests and full reporting of outcomes
☐	☒	Estimates of effect sizes (e.g. Cohen's *d*, Pearson's *r*), indicating how they were calculated

Our web collection on statistics for biologists contains articles on many of the points above.

Software and code

Policy information about availability of computer code

Data collection	DESI-MSI images were acquired using HDImaging v.1.4 software in combination with MassLynx v4.1 (Waters Corporation, Milford, MA);<br>LC-MS data were recorded using Xcalibur v 3.0.63 software (Thermo Scientific);<br>Nano-SIMS images were acquired using Cameca NanoSIMS NS50L Software v 4.4 (Cameca, Gennevilliers, France);<br>Electron microscopy images were collected using Maps software (version 1.1.8.603, Thermo Fisher Scientific);<br>GC-MS data were acquired using MassHunter acquisition software (version 10.0.368)
Data analysis	DESI-MSI data were pre-processed using scripts developed in Python, including Scipy v 1.6.3, MALDIquant v 1.19.3, ComBat (from SVA package v 3.34.0) packages. Colocalisation network analysis and all subsequent statistical modelling were performed in R, v 3.6.2. WGCNA v 1.70-356 was employed to determine a consensus metabolic network.<br><br>The average difference of mean levels of pantothenic acid in each PDX sample and human biopsy between regions of high and low Myc was assessed by a linear mixed effect model fitted with the `glmmTMB` v 1.1.4 package for R.<br><br>METABRIC analysis was done using R, v 3.6.1, the ComplexHeatmap v 2.2.0 and Circlize v 0.4.15 packages.<br><br>The GSVA package v 1.34.0 was applied to infer sample specific MYC pathway activation (The method is freely available as a Bioconductor package for R under the name GSVA at http://www.bioconductor.org.)<br><br>Nano-SIMS images were processed and quantitative data extracted using the OpenMIMS plug-in for ImageJ (https://nano.bwh.harvard.edu/);

Qualitative and quantitative polar LC-MS data analysis was performed using Free Style v 1.5 and Tracefinder v 4.1 software (Thermo Scientific) according to the manufacturer's workflows. For putative annotation CEU Mass Mediator tool was employed.

Qualitative and quantitative apolar LC-MS analyses were performed using Free Style v 1.5 (Thermo Scientific), Progenesis QI v 2.4.6911.27652 (Nonlinear Dynamics) and LipidMatch v 2.02 (Innovative Omics). Radial plot representations were done in R (v 3.6.2) using the R package Volcano3D v 2.08.
MYC IHC signal was deconvoluted using the inbuilt DAB staining deconvolution algorithm of the QuPath software package.

Cell confluency was quantified using Incucyte® S3 Software (Sartorius), v 2021C.

Untargeted metabolic pathway analysis was performed using MetaboAnalyst v 5.0.

Pairwise comparisons were generally carried out using the Student's t-test in Excel or using the R package ggplot2 v 3.4.0 and ggpubr v 0.4.0.

All codes used to carry out the data analysis in the current study including the analysis of mass spectrometry imaging data, MYC pathway activation and IHC data clustering are available at DOI: 10.25418/crick.23925426.

For manuscripts utilizing custom algorithms or software that are central to the research but not yet described in published literature, software must be made available to editors and reviewers. We strongly encourage code deposition in a community repository (e.g. GitHub). See the Nature Portfolio guidelines for submitting code & software for further information.

Data

Policy information about availability of data

All manuscripts must include a data availability statement. This statement should provide the following information, where applicable:

- Accession codes, unique identifiers, or web links for publicly available datasets
- A description of any restrictions on data availability
- For clinical datasets or third party data, please ensure that the statement adheres to our policy

For ChipSeq analysis publicly available data from Sabo et al. were downloaded from the GEO repository with the accession number GSE51011. https://www.ncbi.nlm.nih.gov/geo/query/acc.cgi?acc=GSE51011

The datasets generated during and/or analysed during the current study (including the mass spectrometry imaging data, Immunohistochemistry (IHC) data for PDX samples and human biopsy samples and raw GC-MS data for WM tumours are available at DOI: 10.25418/crick.23925252.

Human research participants

Policy information about studies involving human research participants and Sex and Gender in Research.

Reporting on sex and gender

2 breast cancer biopsies were collected from each female patient.

Population characteristics

Patients from whom the biopsies were collected:
8 - 55 y.o; IDC, Paclitaxel and Herceptin
20 - 62 y.o.; IDC, Letrozole
25 - 84 y.o.; IMC, Paclitaxel and Trastuzumab
28 - 59 y.o.; IDC, Radiotherapy and Letrozole
32 - 57 y.o., IDC, FEC-T
33 - 85 y.o., IDC, Radiotherapy
36 - 68 y.o., mixed IDC and ILC, Radiotherapy and Letrozole
55 - 71 y.o., multifocal IDC, Letrozole
70 - 57 y.o., IDC, Letrozole
80 - 50 y.o., multifocal IDC, AC-Paclitaxel. Radiotherapy.Tamoxifen
81 - 52 y.o., IDC, Radiotherapy
92 - 78 y.o., IDC, Letrozole

Recruitment

Women undergoing breast cancer surgeries were consented to provide their tissue for the study. Data were only obtained on patients who had consented to the utilization of tissue for research. Tumours had to be of a macroscopic size ≥2 cm to allow for adequate research tissue without compromising the clinical diagnosis. Where feasible, tissue was provided from the centre of the tumour from non-necrotic areas.
Based on RNA seq data analysis of a larger sample set, 5 samples with high Myc transcriptional signature (The transcriptomics data were accessed using GSEA querying Hallmark genes, Liberzon A, Birger C, Thorvaldsdóttir H, Ghandi M, Mesirov JP, Tamayo P. The Molecular Signatures Database (MSigDB) hallmark gene set collection. Cell Syst. 2015 Dec 23;1(6):417-425. doi: 10.1016/j.cels.2015.12.004.) and 7 with low MYC transcriptional signature were selected for MYC staining by H&E and DESI-MSI.

Ethics oversight

The institutional review board approved collecting all samples for this study at Imperial College Healthcare National Health Service Trust (Imperial College Healthcare Tissue Bank (ICHTB) HTA licence: 12275 and Tissue Bank sub-collection number SUR-ZT-14-043). REC number : REC Wales approval: 17/WA/0161.

Note that full information on the approval of the study protocol must also be provided in the manuscript.

Field-specific reporting

Please select the one below that is the best fit for your research. If you are not sure, read the appropriate sections before making your selection.

☒ Life sciences ☐ Behavioural & social sciences ☐ Ecological, evolutionary & environmental sciences

For a reference copy of the document with all sections, see nature.com/documents/nr-reporting-summary-flat.pdf

Life sciences study design

All studies must disclose on these points even when the disclosure is negative.

Sample size	All in vitro experiments were routinely carried out with three technical replicates and in three independent biological replicates (unless stated otherwise), which is the minimum number required to perform statistical comparison. In all cases, sample sizes have been reported.<br>For in vivo studies with diet modification group sizes of 5-8 were chosen based on previous experience with these tumour models and metabolism-targeting interventions (Kreuzaler P., et al., 2019, PNAS, 116 (44) 22399-22408. doi.org/10.1073/pnas.190348511; Mendez Lucas A. et al., 2020, Nature Metab., 2(4):335-350. doi: 10.1038/s42255-020-0195-8). For tumour transplant studies with labeled glucose and glutamine samples sizes of 3-4 were chosen due to previous experience (Mendez Lucas A. et al., 2020, Nature Metab., 2(4):335-350. doi: 10.1038/s42255-020-0195-8).<br>For DESI/DEFFI studies of mouse tumours, three tumours of each genotype (WM-high, low, -mix) without infusion, as well as labeled glucose and glutamine infusion were imaged, which was the minimum number required to perform statistical comparison. For PDXs, 6 types of PDXs with 2 independent biological replicates per PDX type were analysed by GC-MS and imaged by DESI/DEFFI.<br>For human breast cancer biopsies, based on RNA seq data analysis of a larger sample set, 5 samples with high MYC transcriptional signature (The transcriptomics data were accessed using GSEA querying Hallmark genes, Liberzon A, Birger C, Thorvaldsdóttir H, Ghandi M, Mesirov JP, Tamayo P. The Molecular Signatures Database (MSigDB) hallmark gene set collection. Cell Syst. 2015 Dec 23;1(6):417-425. doi: 10.1016/j.cels.2015.12.004.) and 7 with low MYC transcriptional signature were selected for MYC staining by H&E and DESI-MSI. Only one section per sample was imaged due to extremely limited availability of the human material.<br>For Nano-SIMS of glucose and glutamine co-infused mice, tissues from three independent mice were analysed, while for Calcium-pantothenate infused mice only two independent mice could be analysed due to technical reasons.
Data exclusions	In vitro: Data was only excluded due to technical failure (e.g. over confluence, at start of experiment, bad melting curve in qRT-PCR, etc). No data was systematically excluded.<br>In vivo studies with diet modification: Only mice with failed tumour grafting or tumour unrelated health issues were excluded.<br>In vivo studies with labeled metabolite administration: Mice with failed metabolite administration due to improper cannulation and mice with failed tumour grafting were excluded.<br>For Nano-SIMS study with Calcium-Pantothenate (dual label 13C and 15N), the 13C trace was recorded, but not analysed, as it failed to show signal above background, due to a higher natural abundance of 13C compared to 15N.
Replication	All experiments were carried out as independent biological replicates, and checked for consistency and equal baseline levels. All attempts at replication were successful.<br><br>Below is a summary of replications of in vitro experiments:<br>Proliferation of 4T1 cells in the presence and absence of PA - 3 technical replicates, the experiment was repeated one more time.<br>Proliferation of 67NR cells with the ectopic expression of SLC5A6 with and without PA - 3 technical replicates, the experiment was reproduced two more times. The representative result is shown due to a difference in seeding density for different repeats.<br>The effect of induction of ectopic MYC expression on the expression of SLC5A6 and PA uptake in 67NR cells - the results are an average of 3 biological replicates.<br>The effect of SLC5A6 overexpression and PA deprivation on the level of citrate in 67NR cells - the result is an average of 3 biological replicates.<br><br>For in vivo experiments, individual animals of control and experimental cohorts are biologically unique -unless mentioned differently, replicate data represents analysis of data/samples from independent replicate animals and is denoted by "n".<br>For the transplant of the WM tumours, different primary tumours were utilised to avoid effects due to genetic drift within a single tumour clone.<br>DESI imaging was reproduced on independent machines at Imperial College London, at the National Physics Laboratory.
Randomization	In vivo studies: To avoid litter-effect, different cell types (e.g. WM-high, -low, -mix) were implanted into mice from different litters in a random fashion. This was also the case in all studies with diet alteration. Before changing the diet, litters were mixed and randomised.
Blinding	In preclinical model experiments, tumour size and mouse weight measurements were blinded as well as treatment regimes. DESI/DEFFI imaging was carried out blind and agnostic to underlying tumour genotypes.

Reporting for specific materials, systems and methods

We require information from authors about some types of materials, experimental systems and methods used in many studies. Here, indicate whether each material, system or method listed is relevant to your study. If you are not sure if a list item applies to your research, read the appropriate section before selecting a response.

Materials & experimental systems

n/a	Involved in the study	
☐	☒	Antibodies
☐	☒	Eukaryotic cell lines
☒	☐	Palaeontology and archaeology
☐	☒	Animals and other organisms
☒	☐	Clinical data
☒	☐	Dual use research of concern

Methods

n/a	Involved in the study	
☒	☐	ChIP-seq
☒	☐	Flow cytometry
☒	☐	MRI-based neuroimaging

Antibodies

Antibodies used

MYC: AbCam, ab32072, clone Y69, Lot GR3377350-24.
SLC5A6: Proteintech 26407-1-AP, polyclonal, Lot 00043017.
b-actin: Merck A3854 (HRP coupled), clone AC-15.
Cleaved caspase 3: Cell Signaling #9664, clone 5A1E, Lot 47.
ATF4: AbCam, ab23760, polyclonal, Lot GR3249618.
PDHE1: AbCam ab110330, clone 9H9AF5.
p70S6K: Cell Signaling, #9202, polyclonal, Lot 14.
p-p70S6K: Cell Signaling, #9234, clone 108D2, Lot 12.
p-S6: Cell Signaling, #4834s, clone D57.2.2E, Lot 8.
S6:⍰ Cell Signaling, #2217, clone 5G10, Lot 10.
HK2: Cell Signaling, #2867S, clone C64G5, Lot 3.
AcSL1: Cell Signalling, #4047, polyclonal.
p-PERK (Thr980): Cell Signaling, #3179, clone 16F8, Lot 20.
PERK: Cell Signaling, #3192, clone C33E10, Lot 11.
p-eIF2α (Ser51): Cell Signaling, #3398, clone D9G8, Lot 1.
eIF2α: Cell Signaling, #5324, clone D7D3, Lot 9.
Secondary antibodies: Anti-rabbit-HRP: GE Healthcare, #NA934-1ML, 1:7.500; Anti-mouse-HRP: Invitrogen, #62-6520, 1:7.500.

Validation

MYC antibody are published in https://doi.org/10.1038/s41467-018-06315-w and validated in our study by ectopic expression in tumours (Fig. 4i) and cells (Ext. Fig. 6c).
SLC5A6 antibody are published in doi: 10.1016/j.mcpro.2022.100217.
b-actin antibody are published in https://doi.org/10.1038/s41467-023-36967-2.
Cleaved caspase 3 antibody are published in https://doi.org/10.1038/s41467-023-39716-7.
ATF4 antibody are validated in DOI: 10.1126/sciadv.abq5575.
PDHE1 antibody are published in doi: 10.1038/s41467-022-28737-3.
p70S6K antibody are published in https://www.nature.com/articles/s41586-022-05652-7.
p-p70S6K antibody are published in https://doi.org/10.1038/s41467-023-36881-7.
p-S6 antibody are published in https://doi.org/10.1038/s41467-023-39261-3.
S6 antibody are published in https://www.nature.com/articles/s41467-023-39261-3.
HK2 antibody are validated by us in https://doi.org/10.1038/s42255-020-0195-8.
AcSL1 antibody are validated in https://doi.org/10.1038/s41467-021-22471-y.
p-PERK (Thr980) antibody are published in https://doi.org/10.1038/s41467-021-25945-1.
PERK antibody are published in https://doi.org/10.1038/s41556-022-00918-8 .

p-eIF2α (Ser51) antibody are published in https://doi.org/10.15252/embj.2022112869.
eIF2α antibody are published in https://doi.org/10.15252/embj.2022112869.

Eukaryotic cell lines

Policy information about cell lines and Sex and Gender in Research

Cell line source(s)

4T1: The Francis Crick institute, cell services
67NR: The Francis Crick institute, cell services
67NR-tet-cMYC-IRES-eGFP cells were generated by Peter Kreuzaler in the laboratory of Gerard Evan and have been published.

Authentication

The cell lines were authenticated using a standard protocol for identification of mouse cell lines using STR (Short Tandem Repeat) Profiling. The profile is compared back to any available on commercial cell banks (ATCC, Cellosaurus, etc). Species is confirmed by using a primer system, based on the Cytochrome C Oxidase Subunit 1 gene from mitochondria.

Mycoplasma contamination

All cells were tested negative for Mycoplasma by the Facilities at the Francis Crick institute prior to utilisation

Commonly misidentified lines
(See ICLAC register)

None

Animals and other research organisms

Policy information about studies involving animals; ARRIVE guidelines recommended for reporting animal research, and Sex and Gender in Research

Laboratory animals	Only female mice were used in this study.<br>To generate spontaneous non-recombined tumours as a source of biclonal tumours, Rosa26-CAG-lox-STOP-lox-MycERT2/ Rosa26-mTmG/MMTV-Wnt1 mice were used. The transgenes used to generate the cross were on the following backgrounds: MMTV-Wnt1: FVB/N; Rosa26-mTmG: C57BL/6J, and Rosa26-CAG-lox-STOP-lox-MycERT2:Balb/c. The tumours arose spontaneously between 4 and 8 months of age.<br>WM tumour transplants were generated in female NOD/Scid micemice at 6-8 weeks of age.<br>The PDX panel of mice was transplanted into female NSG mice (NOD.Cg-Prkdcscid Il2rgtm1Wjl/SzJ) at the age of 5 weeks and took 2-10 months to form.<br>WM tumour transplants and HCI002 transplants during diet modification were done into female NOD SCID mice (NOD.CB17-Prkdcscid/NCrCrl); mice were placed either on control or PA-deficient diet at the age of 7 weeks and tumour cells were grafted 5 weeks after.<br>67NR control and SLC5A6 overexpressing cell lines were transplanted into female Balbc/C J mice at the age of 7 weeks.
Wild animals	No wild animals were used in the study.
Reporting on sex	As the study was done on breast cancer, only female mice were utilised.
Field-collected samples	No field collected samples were used in the study.
Ethics oversight	All procedures and animal husbandry were carried out in accordance with the UK Home Office, under the Animals (Scientific Procedures) Act 1986, and the Crick Animal Welfare and Ethical Review Body (AWERB), which is delivered as part of the Biological Research Facility (BRF) Strategic Oversight Committee (BRF-SOC), under the Project licence number P609116C5.

Note that full information on the approval of the study protocol must also be provided in the manuscript.
