# Supplementary material for: Vitamin B5 supports MYC oncogenic metabolism and tumor progression in breast cancer
Source: Nat Metab. 2023 Nov 9;5(11):1870–86. doi: 10.1038/s42255-023-00915-7 (PMC10663155; doi:10.1038/s42255-023-00915-7)
Supplement: Supplementary file 8 — Uncut western blots for Fig. 4j,l. [file 42255_2023_915_MOESM8_ESM.pdf]

Fig 4j

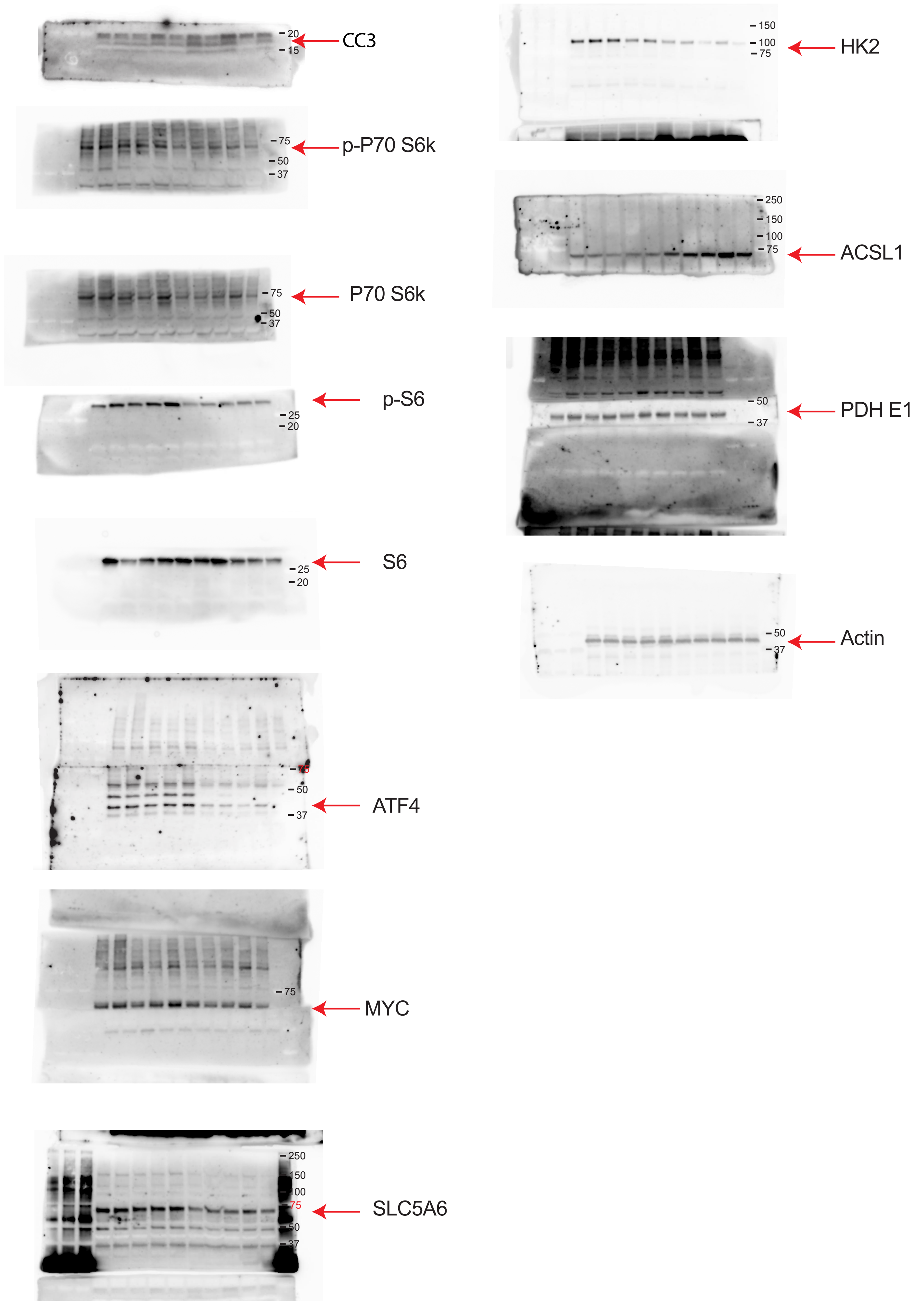
CC3
20
15
p-P70 S6k
75
50
37
P70 S6k
75
50
37
p-S6
25
20
S6
25
20
ATF4
75
50
37
MYC
75
SLC5A6
250
150
100
75
50
37
HK2
150
100
75
ACSL1
250
150
100
75
PDH E1
50
37
Actin
50
37

Fig 4l

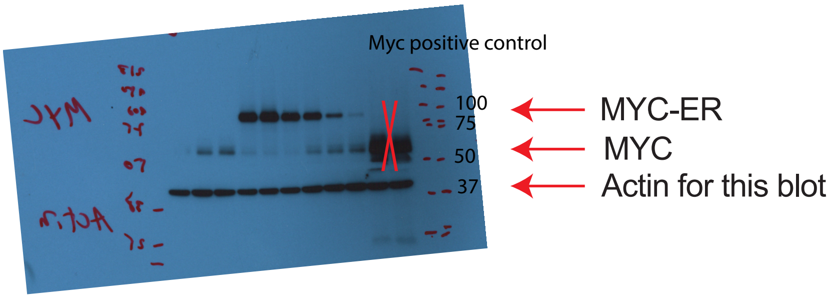

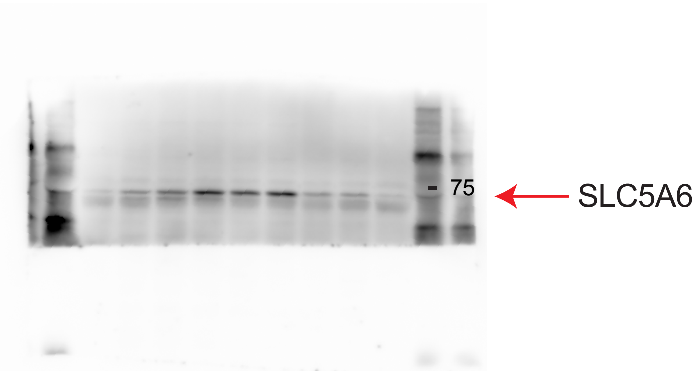

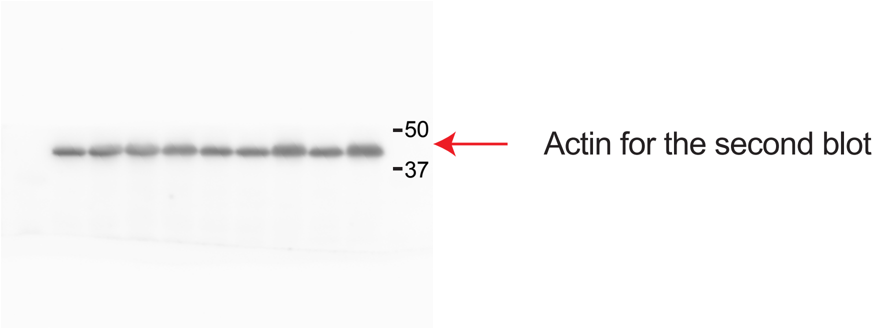

The same lysates where used for these runs, but once run with a positive control for Myc and once without.
Top blot was converted to black and wite in the manuscript
