# Supplementary figures and images for: Vitamin B5 supports MYC oncogenic metabolism and tumor progression in breast cancer
Source: Nat Metab. 2023 Nov 9;5(11):1870–86. doi: 10.1038/s42255-023-00915-7 (PMC10663155; doi:10.1038/s42255-023-00915-7)

Ex Fig 6e Western Blots

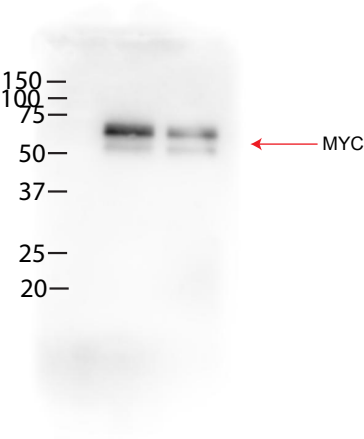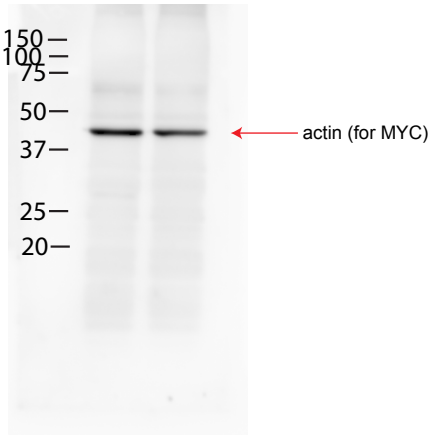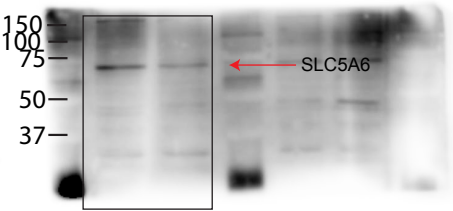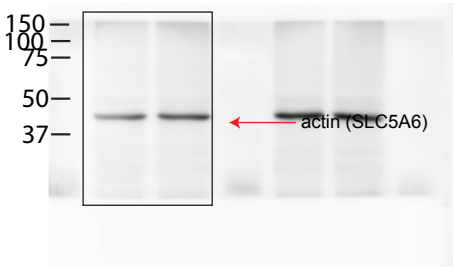

Ex Fig 6c Western Blots

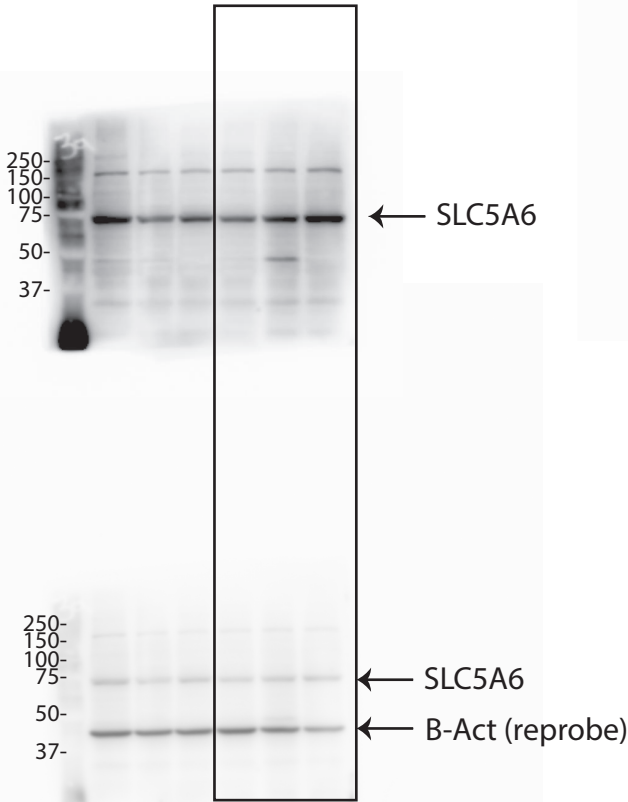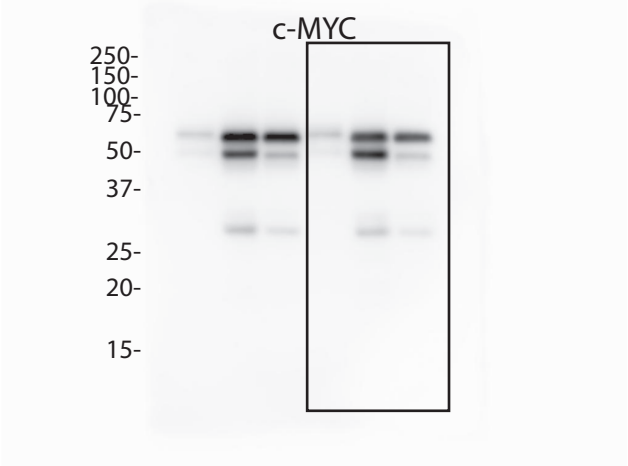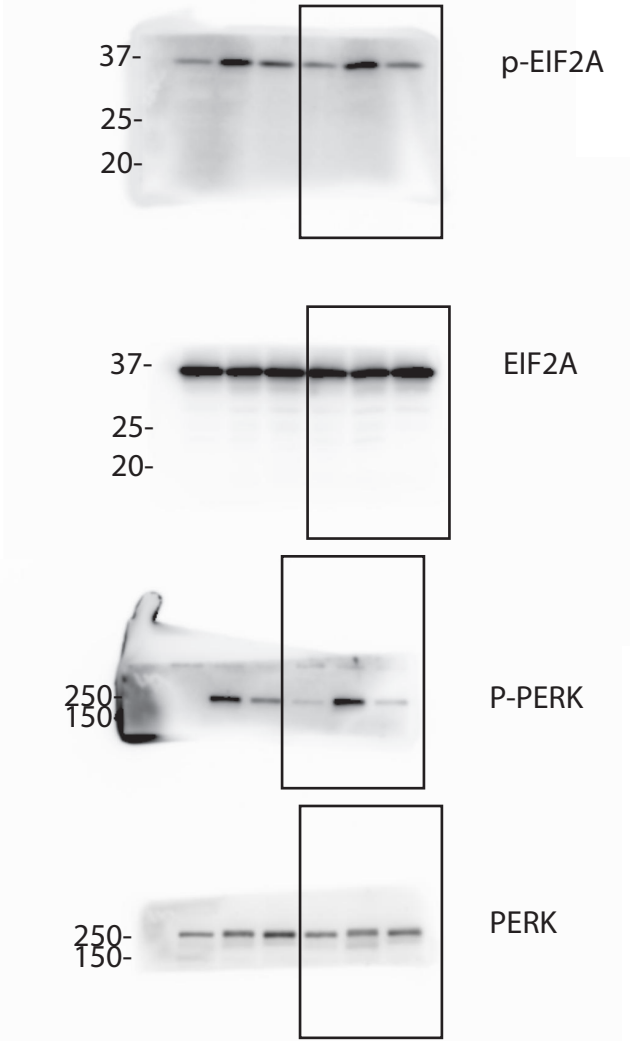

Ex Fig 6a Western Blots

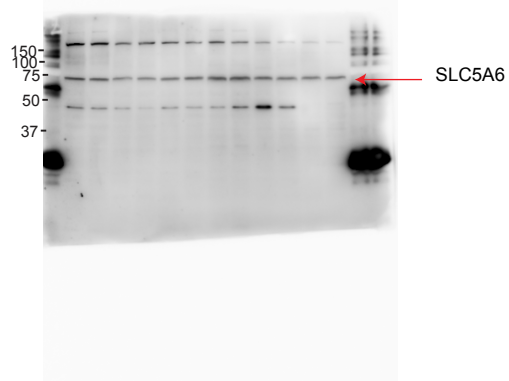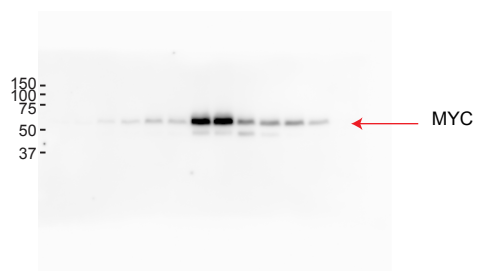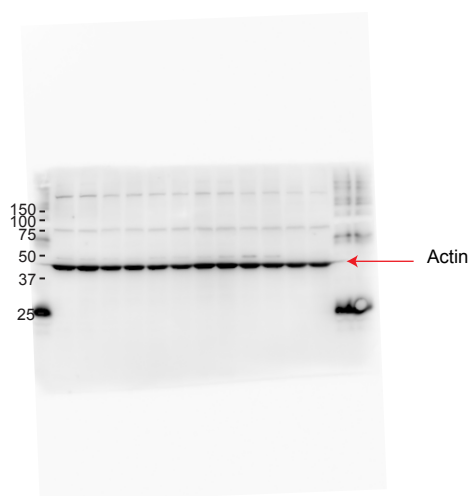

Supplement: Supplementary file 13 — Uncut western blots for Extended Data Fig. 6a,c,e. [file 42255_2023_915_MOESM13_ESM.pdf]
